# A comprehensive comparison of web-based tools for amplicon-metagenomic analysis

**DOI:** 10.3389/fmicb.2025.1711000

**Published:** 2026-01-06

**Authors:** Yuhe Kan, Xiao-Yu Ma, Yi-Lu Wang, Baiye Sun, Shoule Wang

**Affiliations:** 1 College of Biology and Oceanography, Weifang University, Weifang, Shandong, China; 2 College of Food and Drug, Weifang Vocational College, Weifang, Shandong, China; 3 Shandong Institute of Pomology, Shandong Academy of Agricultural Sciences, Tai’an, China

**Keywords:** amplicon sequencing, comparison, functional modules, microbiome profiling, web-based tools

## Abstract

Amplicon sequencing provides a suitable approach for microbiome profiling, supported by a variety of R-based and web-based tools. In this review, we systematically evaluated eight freely accessible web-based tools suitable for users without scripting experience, comparing their performance across modules including alpha and beta diversity, taxonomic composition, differential comparison, network and correlation analysis, functional profiling, machine learning, tree-plot and user experience. While all tools exhibit limited data filtering and normalization options, performance varied considerably across modules. Mian and MicrobiomeAnalyst 2.0 excelled in alpha diversity analysis and taxonomic composition analysis, METAGENassist outperformed others in beta diversity, and MicrobiomeAnalyst 2.0 achieved the highest score in differential comparison and functional analysis. Namco and Mian outperform in network analysis and correlation analysis, respectively. Machine-learning functions were comparable across animalcules, MicrobiomeAnalyst 2.0 and METAGENassist, with the best treeplot visualization in animalcules and MicrobiomeAnalyst 2.0. And, user experience was highest for animalcules and Mian. Overall, MicrobiomeAnalyst 2.0 achieving the highest overall performance, followed by Mian and Namco. Several limitations among evaluated tools include inconsistent accessibility, diverse input data formats, restricted feature sets, and incomplete retention of key information in exported figures. Future development should integrate preprocessing, interactive visualization and figure export, alongside advanced statistical methods, multi-omics integration and meta-analytical capabilities, to enhance flexibility, reproducibility and interpretability. This comprehensive assessment provides a practical reference for researchers in selecting the most suitable web-based tools for specific microbiome analysis tasks, highlighting the importance of both module-specific performance and overall tool capabilities.

## 1 Introduction

The microbiota plays important roles on environment protection (Zhu et al., 2022), bioenergy (Koch et al., 2014), agricultural production (Suman et al., 2022), food (Balasubramanian et al., 2024) and human health (Ross et al., 2024). The microbial diversity and community structure in host have a high relationship with host phenotypes. For example, soil microbiomes can enhance plant nutrition and abiotic stress management by activating plant defense systems, enzyme production, protein regulation and the production of growth factors (Adeleke et al., 2024). Hence, the systematic microbial profiles, such as microbial diversity, community structure and functionality, are required for better understanding the complex interactions between microorganisms and their hosts.

In order to study the microbial profiles, high-throughput sequencing technologies, such as Illumina (Modi et al., 2021), PacBio and Oxford Nanopore Technologies (ONT) (Lang et al., 2020), as well as recently developed BarBIQ method (Barcoding Bacteria for Identification and Quantification) (Jin et al., 2022; Jin et al., 2024), which enables taxonomic classification of individual bacterial cells, can provide powerful alternatives to traditional culture-based methods. Traditional cultivation methods are labor-intensive, time-consuming and costly, and thus a large proportion of microorganisms remain uncultured to date (Kim, 2023). Currently, the phylogenetic marker-gene amplicon sequencing approach, e.g., 16S rRNA gene amplicon sequencing, regarded as a fast and cost-effective approach, is widely used for microbial analysis (Zhang et al., 2023). To meet the rapidly growing demand for marker gene sequencing data analysis, the tools or bioinformatic pipeline for data processing and data visualization of microbiome data are emerged in recent years. Until now, many popular tools are available, such as mothur (Schloss et al., 2009), QIIME2 (Bolyen et al., 2019) and R packages including phyloseq (McMurdie and Holmes, 2013), animalcules (Zhao et al., 2021), MicrobiomeStat (Zhou et al., 2022), MicrobiomeAnalyst (Dhariwal et al., 2017; Chong et al., 2020), MicrobiomeAnalyst 2.0 (Lu et al., 2023), EasyAmplicon pipeline (Liu et al., 2023), microeco (Liu C. et al., 2021a), microViz (Barnett et al., 2021), microbiomeSeq (Ssekagiri et al., 2017), microbiomeMarker (Cao et al., 2022), EasyMicroPlot (Liu et al., 2022) and MicrobiotaProcess (Xu et al., 2023), etc. Although R program is a powerful approach for analyzing microbial data, their application can be challenging for users without coding experience. Thus, the web-based tools are allowed for beginners with limited experience in command-line interfaces to perform microbiome analysis for their microbiome data, such as comprehensive meta-analysis, statistical analysis and interactive visualizations.

However, variations in the selection of features or analytical options within web-based tools, such as statistical methods or algorithms, can yield different conclusion and potentially lead to spurious findings. Recently, a review of the current state of freely accessible web tools for the analysis of 16S rRNA sequencing was reported (Ibal et al., 2022). However, the detail and comprehensive comparisons among newly published web-based tools was not reviewed. Hence, the comparison of the web-based tools for amplicon metagenomic analysis need to be conducted. Firstly, we provide a detail comparison of general categories including data summary, data filtering and data normalization, as well as statistical method and algorithms in the process of downstream analysis. Secondly, we compare and evaluate the performance of selected web-based tools. Lastly, we highlight the current limitations and outline suggestions and future perspectives for microbiome analysis using web-based tools. Our review will guide beginner who have less experience in using command-line interfaces to select suitable online-based tools for microbiome analysis, which contribute to better characterize and understand the microbial community structure in their study.

## 2 General information of selected web-based tools

**Figure 1:**
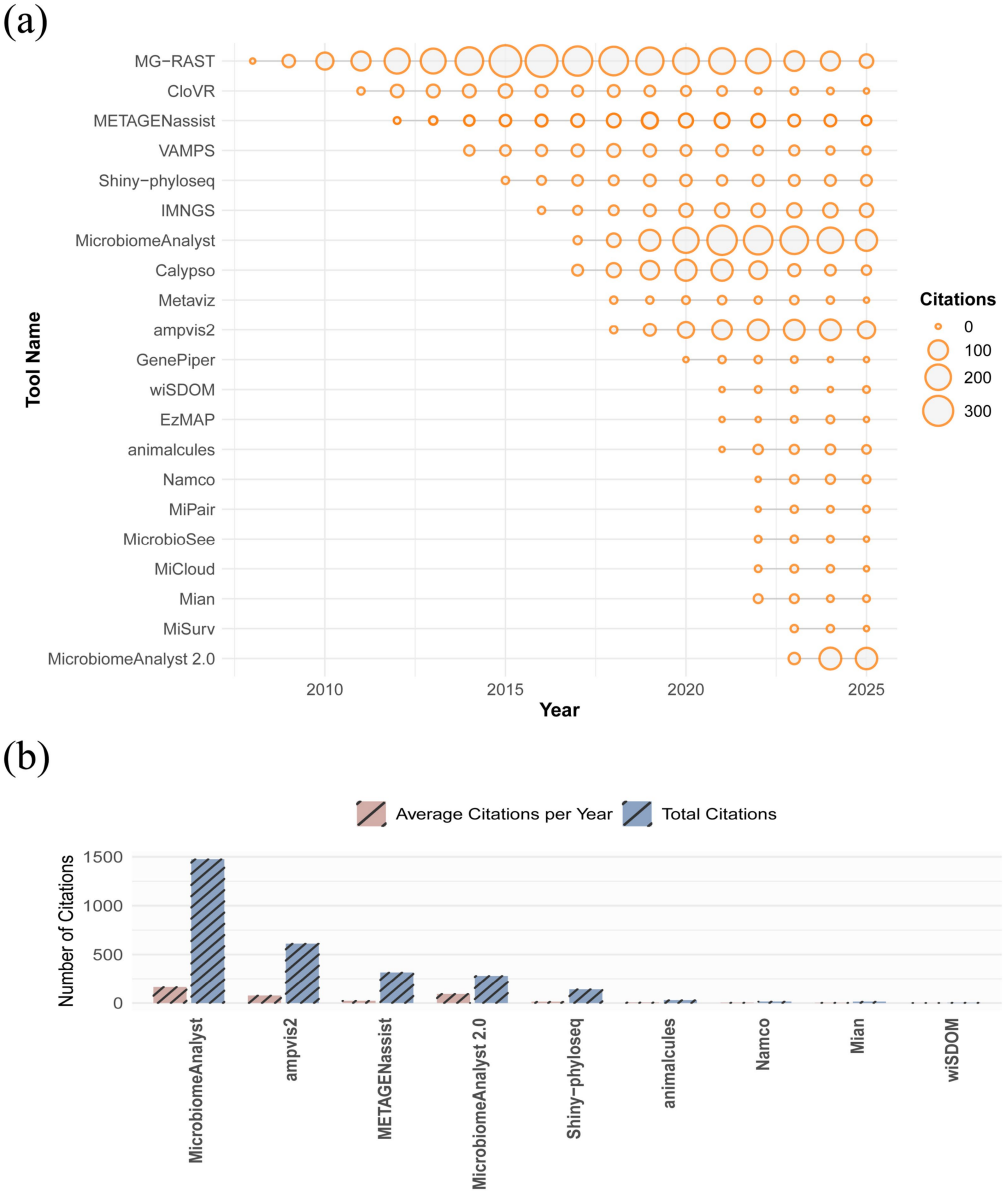
**(a)** The citation timeline of the 20 tools; **(b)** Comparison of total citations and average citations per year for the eight selected tools. MicrobiomeAnalyst and MicrobiomeAnalyst 2.0 represent different versions of the same tool.

**Figure 2:**
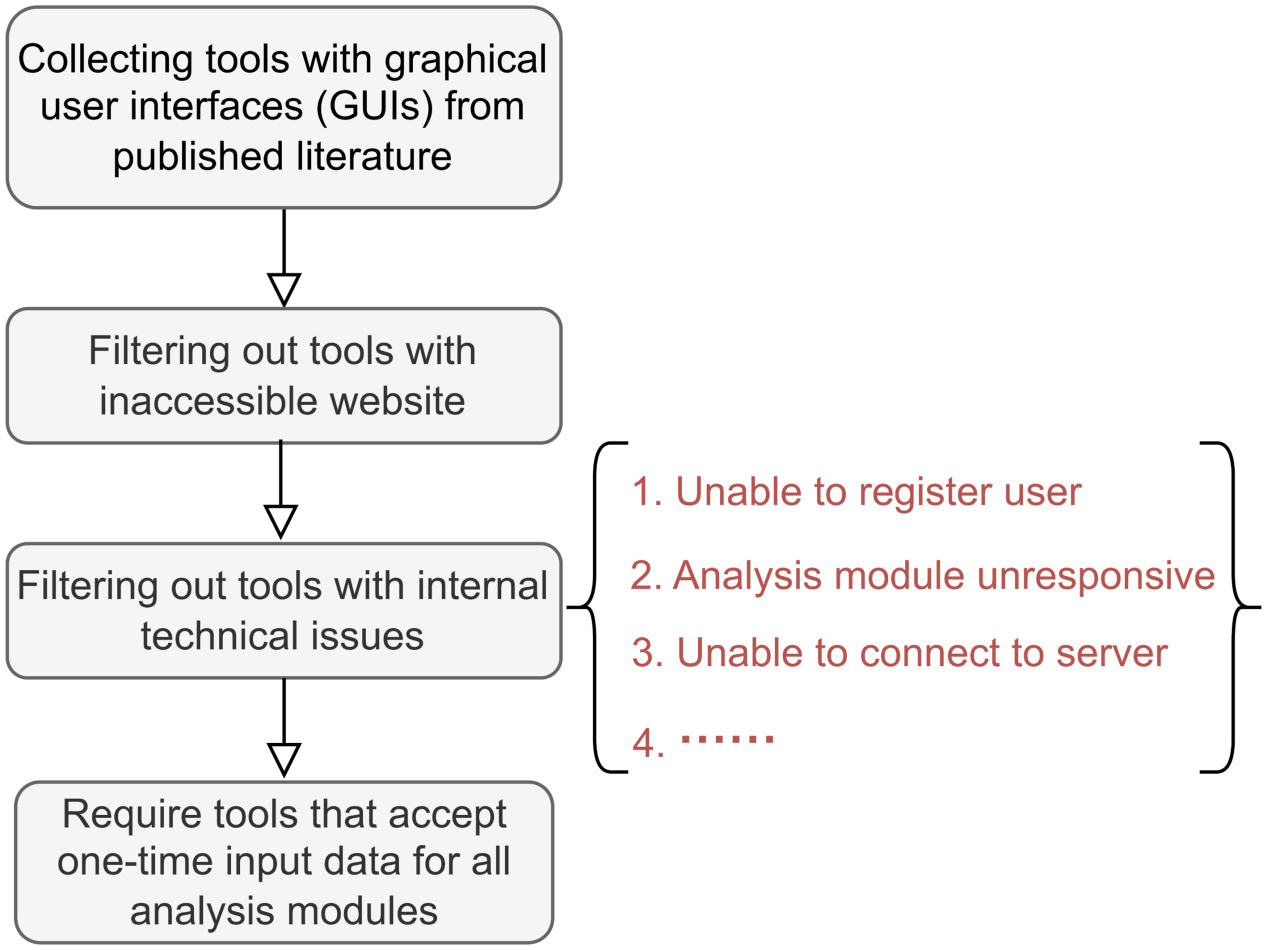
Workflow for selecting tools for comparative analysis.

Through an extensive literature review, a total of 20 web-based tools for amplicon-metagenomic analysis were identified by us. To illustrate the year-by-year usage, the citation timeline was shown in Figure 1a. MG-RAST, developed in 2008, represents one of the earliest online platforms, with citations peaking around 2015 and gradually declining thereafter. Subsequently, MicrobiomeAnalyst (First released in 2017 and updated to version 2.0 in 2023) gained widespread popularity as a comprehensive and user-friendly online tool. Overall, the citation trends indicate a marked acceleration in the development of online tools after 2015, reflecting both the rapid expansion of bioinformatics resources and the increasing demand for accessible online platforms. We tested all identified tools using an example dataset (see Supplementary file S1 for details on input data preparation for each tool). Tools meeting the following criteria were selected for comparative analysis (Figure 2): (1) availability of a graphical user interfaces (GUIs); (2) online accessibility with active and valid URLs; (3) absence of major technical issues, i.e., server failures, registration errors or connection problems; and (4) support for a single input dataset applicable across analysis modules, rather than requiring separate input files for each analysis module. Based on these criteria, eight representative web-based tools—Shiny-phyloseq, MicrobiomeAnalyst 2.0, ampvis2, wiSDOM, animalcules, Namco, METAGENassist and Mian—were selected for comparative analysis. Tools excluded from further evaluation, along with the reasons for exclusion, are listed in Supplementary Table S1. According to citation statistics (Figure 1b), The top3 web-based tools in terms of total citations and average citations per year were MicrobiomeAnalyst (2.0), ampvis and METAGENassist, suggesting that MicrobiomeAnalyst (2.0) remains one of the most influential platforms in the field. These eight tools can therefore be considered representative in the context of microbiome analysis, given their recent updates, comprehensive analytical modules (e.g., Mian, Namco, and MicrobiomeAnalyst 2.0), and relatively high scientific impact.

## 3 Input data preparation

### 3.1 Input data requirement

Prior to downstream analysis, input data must be uploaded. Most tools support various data types, with count tables and BIOM format being the most commonly used (Supplementary Table S2). However, different tools have specific requirements for count tables. For instance, ampvis2 requires feature tables in which the last seven columns contain taxonomic annotations from kingdom to species. The detailed requirements for each tool are available in their online manuals and are not discussed here. The R scripts for converting a phyloseq object into the appropriate standalone input formats required by the evaluated tools were provided in Supplementary file S1 and Supplementary Figure S1. We recommended that researchers use the R script to efficiently prepare their own datasets. Furthermore, we strongly advocate for the standardization of input data formats across web-based tools, i.e., ideally allowing acceptance of phyloseq objects, to minimize data conversion efforts and streamline analysis across platforms.

### 3.2 Data summary

After uploading the input data, the data summary generated by tools provides users with general information (Supplementary Table S3), facilitating the detection of abnormal data and supporting the selection of appropriate parameters or options for data filtering and data normalization. Most tools report basic statistics including number of samples, covariates and features, while additional summary outputs vary across platforms. For instance, animalcules and MicrobiomeAnalyst 2.0 provide detailed count-based summaries across samples, including sample mean, median, minimum and maximum counts, and animalcules further reports mean and median counts at the feature level. Moreover, animalcules, Mian and Namco allow users to generate and download feature tables aggregated at different taxonomic levels for downstream analysis. Namco also reports feature counts below thresholds of 0.25% relative abundance and 10% prevalence, providing users with greater flexibility when assessing data quality.

### 3.3 Data filtering

Quality-filtering is essential for minimizing biases or avoiding inaccuracies in downstream analysis (Bokulich et al., 2013). In practice, filtering of the abundance matrix typically involves two types: sample filtering and feature filtering (Zhou et al., 2023). Sample filtering focuses on removing low-quality samples resulting from poor sample or DNA quality, as well as technical errors. Eliminating such samples is critical for ensuring an accurate representation of the microbial community while preserving sufficient statistical power. Samples with markedly smaller library sizes compared with the rest of the dataset or within specific covariate groups are generally candidates for removal. It is noteworthy that MicrobiomeAnalyst 2.0 performs sample filtering strictly based on sample names (sampleID), rather than metadata-defined groups. Feature filtering aims to remove non-informative taxa with low abundance, low prevalence or minimal variance across samples. Available filtering options vary widely among tools and may include thresholds based on mean read count or relative abundance, prevalence, the removal of singletons, or variance-based criteria. Most tools offer only a subset of these options, whereas MicrobiomeAnalyst 2.0, METAGENassist and Namco provide more extensive and flexible filtering settings (Supplementary Table S3). For variance filtering, MicrobiomeAnalyst 2.0, METAGENassist and Namco support this function, but MicrobiomeAnalyst 2.0 and METAGENassist offers more specific sub-options (inter-quantile range, standard deviation and coefficient of variation) than Namco that without sub-options. Beyond abundance- and prevalence-based filtering, *decontam* is a widely recommended method for identifying and removing contaminant taxa that show inverse correlations with sample DNA concentration (Davis et al., 2018). Among the reviewed tools, only Namco incorporates *decontam*. Any taxa strongly associated with identified contaminants or batch factors should be excluded from downstream analysis (Zhou et al., 2023). Despite the availability of multiple filtering strategies, selecting appropriate thresholds require careful consideration. Overly stringent filtering may introduce bias or discard biologically meaningful rare taxa, while overly lenient criteria may retain noise. Currently, no standardized guidelines exist across web-based platforms, and each tool relies on its own preferred methods. Thus, filtering criteria is inherently subjective but should be guided by the study design and biological understanding of the microbial community. Therefore, researchers are encouraged to evaluate the rationale behind their filtering criteria to avoid inadvertently excluding important and informative features.

### 3.4 Data normalization and statistical test

Due to zero inflation, compositionality and overdispersion in microbiome abundance data (Lutz et al., 2022), appropriate data normalization is essential prior to downstream analysis. Among these tools, MicrobiomeAnalyst 2.0, Shiny-phyloseq, animalcules, METAGENassist and Namco shared less common methods, with MicrobiomeAnalyst 2.0 offering the widest selection of normalization options (Supplementary Table S3). Notably, Namco provides the option for spike-in normalization, which enables absolute quantification of microbial abundance by incorporating known amounts of external spike-in controls (Zemb et al., 2020). This functionality is not available in the other tools, some of which do not offer any normalization methods at all. Additional normalization strategies for microbiome abundance data have been comprehensively summarized in recent review article (Xia, 2023; Zhou et al., 2023). The performance of normalization is highly sensitive to the chosen methods, sample size and the presence of rare features. Therefore, researchers must carefully select a normalization strategy that aligns with the characteristics and goals of their specific dataset to ensure robust and reliable downstream analysis. Statistical tests applied to normalized data constitute the core analytical framework in microbiome research, underpinning tasks such as identifying taxa that differ across phenotypes, characterizing microbiom–covariate associations, and inferring microbial interaction networks. In our review, tools implementing statistical tests generally apply multiple testing corrections to control false discovery rates and improve the robustness of findings. A review article is strongly recommended for a more comprehensive understanding of their broader applications in microbiome data analysis (Lutz et al., 2022), and the corresponding statistical methodologies will be mentioned in subsequent modules.

## 4 Data visualization

Data visualization allows researchers to intuitively explore complex data patterns and trends, thereby uncovering potential biological insights. Building on the practical guidelines for amplicon and metagenomic analysis provided by Liu Y. X. et al. (2021b), and considering the visualization modules implemented available tools (Supplementary Table S4), we classified the visualization approaches into nine major analytical modules (Supplementary Table S5): alpha diversity, beta diversity, taxonomic composition, differential comparison, correlation analysis, network analysis, machine learning, treemap and functional prediction. In the following sections, each functional module is evaluated using example datasets (the “caporaso” dataset from the microbiomeMarker package), followed by an in-depth discussion of the corresponding analytical approaches. The “Raw sequencing processing” module, implemented only in Namco and MicrobiomeAnalyst 2.0, was excluded from this review because it falls outside the intended scope, which focuses on comparing tools designed for downstream visualization.

### 4.1 Case study dataset

In this review, the *caporaso* dataset (a phyloseq object) included in the microbiomeMarker package was used as a representative example to evaluate and compare performance of multiple tools. The dataset contained feature table, taxonomy table, metadata and phylogenetic tree file. The dataset contains a total of 34 samples, 3,426 features and 8 sample variables, encompassing a broad range of habitat-related metadata, including SampleType, Year, Month, Day, Subject, ReportedAntibioticUsage, DaysSinceExperimentStart and Description. Researchers can select appropriate tools according to their research requirement to explore the fundamental properties of their data, thereby obtaining useful guidance for data filtering and data normalization. To emphasize differences in functional modules across the tools, no data filtering was applied, based on two considerations: (1) the absence of standardized filtering procedures across all tools, and (2) the objective of comparing tools’ performance rather than analytical outcomes. In the following sections, we compare the data visualization across the eight selected tools.

### 4.2 Systematic performance comparison of the evaluated tools

To systematically evaluate the performance of the selected tools across different functional modules, a weighted scoring framework was established based on key assessment indicators (Supplementary Table S6). Each module (e.g., alpha diversity, taxonomic composition, network analysis) and sub-module (e.g., boxplot, rarefaction curve and rank abundance curve in alpha diversity) were assigned a specific weight reflecting its relative importance. The raw scores obtained for each sub-module were then normalized to a 0–1 scale by dividing them by the maximum weighted score of that module, as illustrated in Equation 1.


$$s_{\mathrm{sub}-\text{module}}=\sum_{i=1}^{n}(w_{i}\times \frac{s_{i,\mathrm{raw}}}{s_{i,\max-\mathrm{raw}}})$$ (1)

where $s_{i,\mathrm{raw}}$ is the raw score, $s_{i,\max-\mathrm{raw}}$ is the maximum score, $w_{i}$ is the weight of criteria.

The module score was calculated as Equation 2:


$$s_{\text{module}}=\frac{\sum_{i=i}^{n}s_{i,\mathrm{sub}-\text{module}}}{\sum_{i=1}^{n}s_{i,\max-\text{submodule}}}$$ (2)

where $s_{i,\mathrm{sub}-\text{module}}$ is the score of sub-modules, $s_{i,\max-\text{submodule}}$ is the maximum score of sub-modules.

### 4.3 Alpha diversity

Alpha diversity characterizes species richness and/or evenness within a sample and is commonly evaluated using statistical comparisons across samples or experimental groups. In Supplementary Table S7, four primary visualization approaches for alpha diversity analysis were identified, including rarefaction curve, venn diagram and boxplot, as reported in the review by Liu Y. X. et al. (2021b). In addition, we considered the rank abundance curve to be an visualization method of alpha diversity, as it provides valuable insights into species evenness and abundance distribution. A comprehensive comparison of the corresponding tool-specific features is presented below. Rarefaction curves can be generated by wiSDOM, Mian, Namco and MicrobiomeAnalyst 2.0. As illustrated in Supplementary Figure S2, Mian, Namco and MicrobiomeAnalyst 2.0 enable the assessment of sequencing depth at individual-sample level. In contrast, wiSDOM provides only an aggregated overview across all samples, limiting the ability to detect inadequate sequencing depth for specific samples within groups. Furthermore, both MicrobiomeAnalyst 2.0 and Mian support group-level visualization through color-coded curves, enabling rapid comparison of sequencing depth among groups. While figures exported from Namco lack explicit sample and group annotations, limiting their suitability for publication. Both Mian and Namco offer interactive visualization exploration of sample information, helping to mitigate curve overlap in large datasets. By contrast, the static displays in MicrobiomeAnalyst 2.0 often exhibit overlapping sample labels, which hinder the accurate identification of individual samples. The rank abundance curve is generated exclusively by wiSDOM (Supplementary Figure S3), allowing abundance distribution to be visualized according to selected metadata variables. However, the fixed step size of the x-axis imposes constraints on data interpretation, potentially reducing the resolution for detecting low-abundance taxa and limiting cross-group comparisons of species distribution patterns.

**Figure 3:**
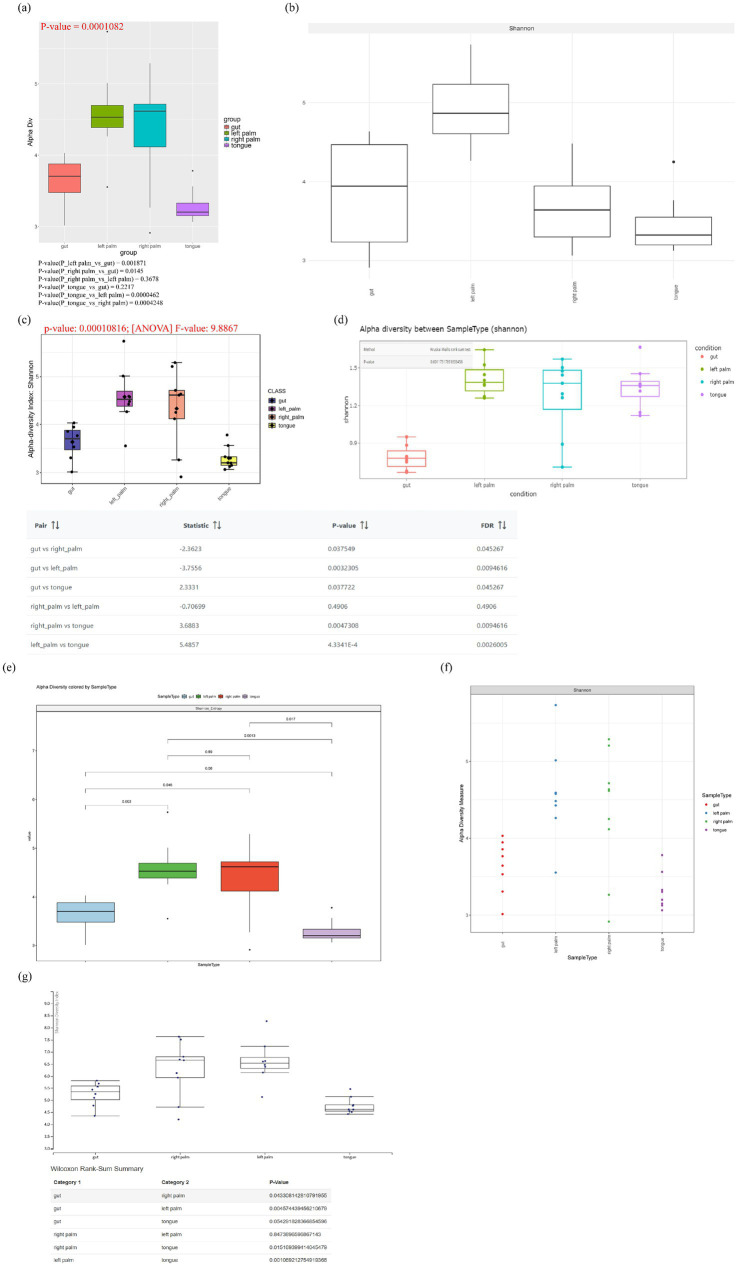
Boxplots of alpha diversity based on the Shannon index, generated by the following tools: **(a)** wiSDOM, **(b)** ampvis2, **(c)** MicrobiomeAnalyst 2.0, **(d)** animalcules, **(e)** Namco, **(f)** Shiny-phyloseq, and **(g)** Mian.

**Figure 4:**
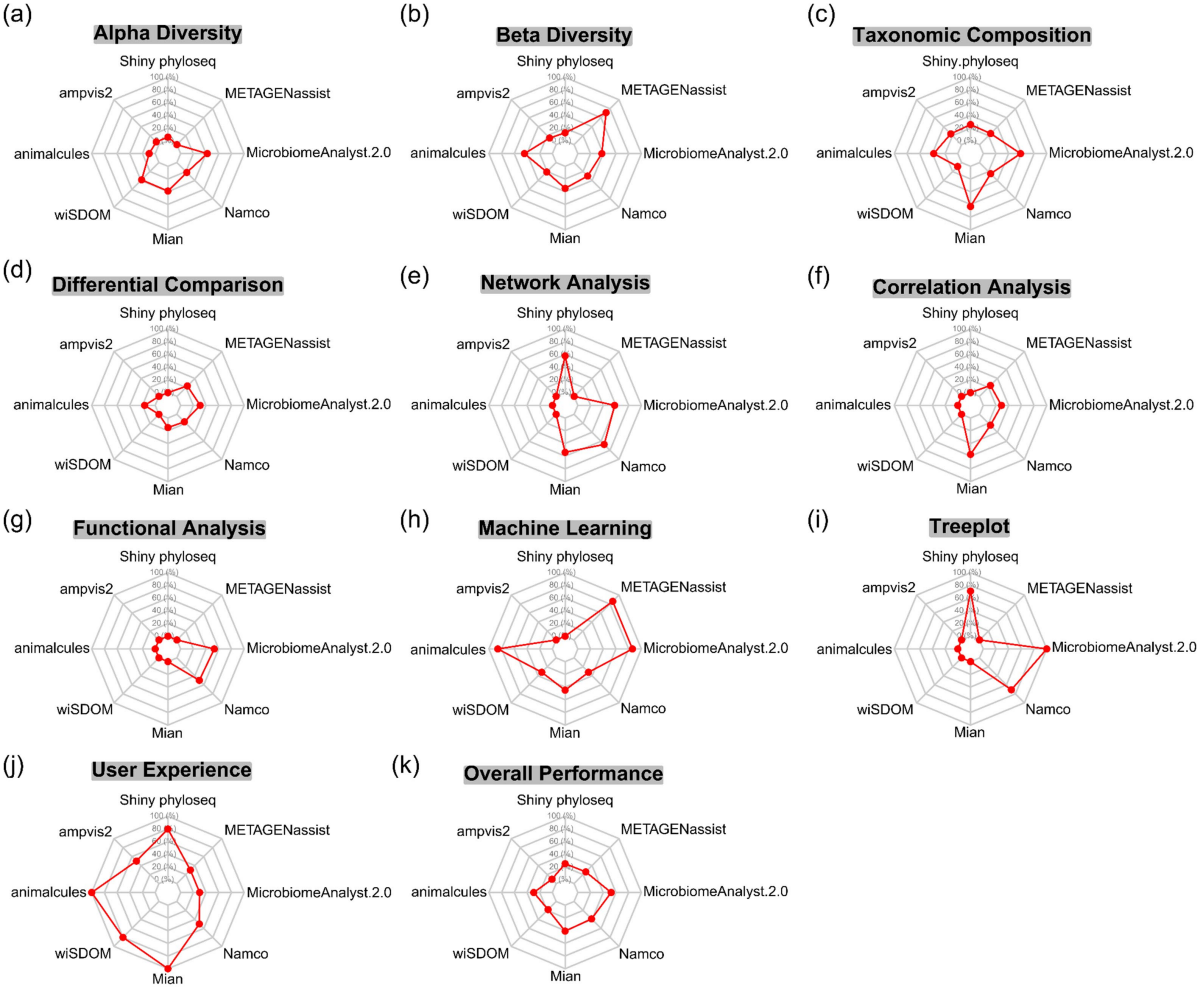
Radar plot showing the performance comparison of tools across modules including **(a)** Alpha diversity, **(b)** Beta diversity, **(c)** Taxonomic composition, **(d)** Differential composition, **(e)** Network analysis, **(f)** Correlation analysis, **(g)** Functional analysis, **(h)** Machine learning, **(i)** Treeplot, **(j)** User experience, and **(k)** Overall performance.

**Figure 5:**
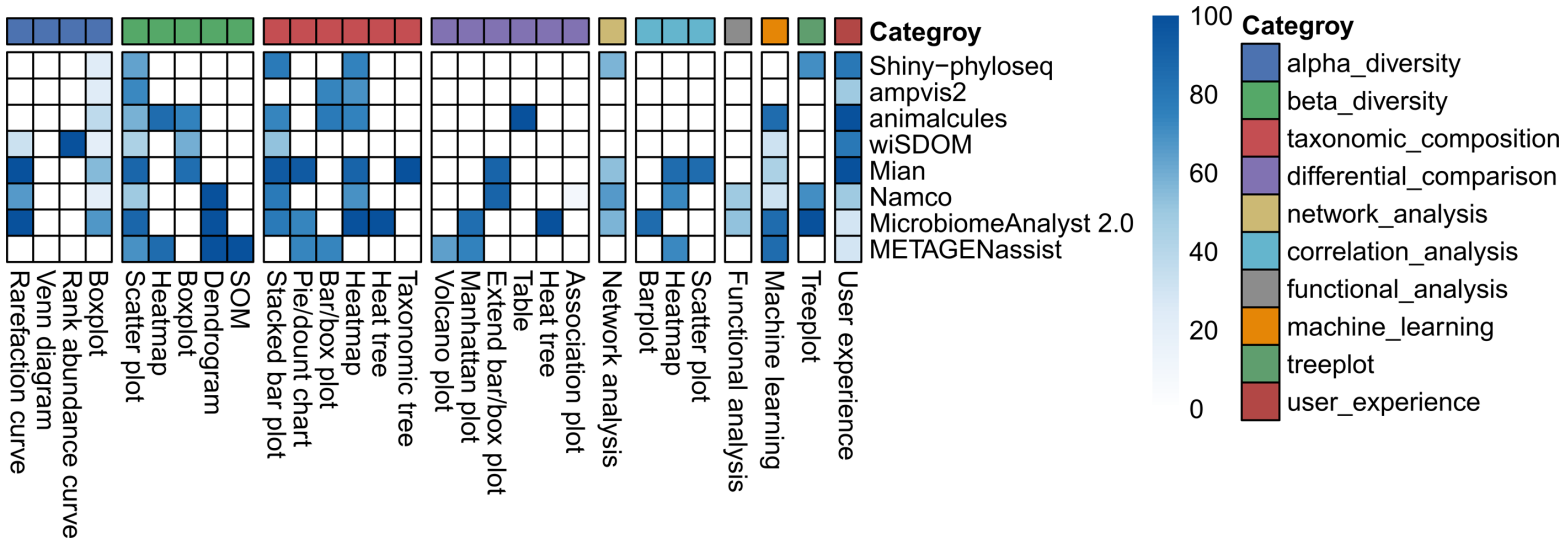
Heatmap showing the sub-module-level comparison across tools.

Boxplots are widely used visualization method in alpha diversity analysis, typically derived from predefined diversity metrics and accompanied by appropriate statistical test. In Supplementary Table S7, 22 diversity metrics were classified into four categories (richness, dominance, phylogenetics and information) according to Cassol’s report (Cassol et al., 2025). Three additional metrics including Gini Simpson, inverse Simpson and effective Shannon entropy that were not explicitly reported in Cassol’s study, were also incorporated into the corresponding categories because they represent synonymous or closely related measures that share substantial similarity in their mathematical formulations. Although each tool provides its own set of diversity metrics and statistical methods, there is considerable overlap among them, particularly in the commonly used indices such as observed richness, Chao1, ACE, Shannon and Simpson. A comprehensive description of these diversity metrics, including guidance on result interpretation and recommended usage, can be found in the review by Cassol et al. (2025). Clearly, tools with fewer diversity metrics offer a narrower basis for interpreting microbial community patterns, with Shiny-phyloseq providing the most and animalcules the fewest. Except for Shiny-phyloseq and ampvis2, all other tools support statistical testing for group-level comparisons, although wiSDOM does not explicitly specify the statistical methods employed. Shiny-phyloseq, ampvis2, wisdom and Namco perform alpha-diversity at an unspecified taxonomic level, whereas animalcules restricts its input count table to taxonomic levels from kingdom to species. For consistent comparison across tools, the Shannon diversity index was selected for subsequent visualization (Figure 3). wiSDOM, MicrobiomeAnalyst 2.0, Namco and Mian can support pairwise statistical comparisons, whereas animalcules reports only an overall *p* value without conducting post-hoc analysis. Among these tools, MicrobiomeAnalyst 2.0 is the only platform that implements multiple testing correction using the false discovery rate (FDR). This distinction underscores the critical importance of implementing appropriate statistical tests for robust interpretation. In the module-level evaluation of alpha diversity, Mian and MicrobiomeAnalyst 2.0 achieved higher scores for alpha diversity analysis (Figure 4a), with notably stronger performance in generating rarefaction curves and box plots (Figure 5).

### 4.4 Beta diversity

**Figure 6:**
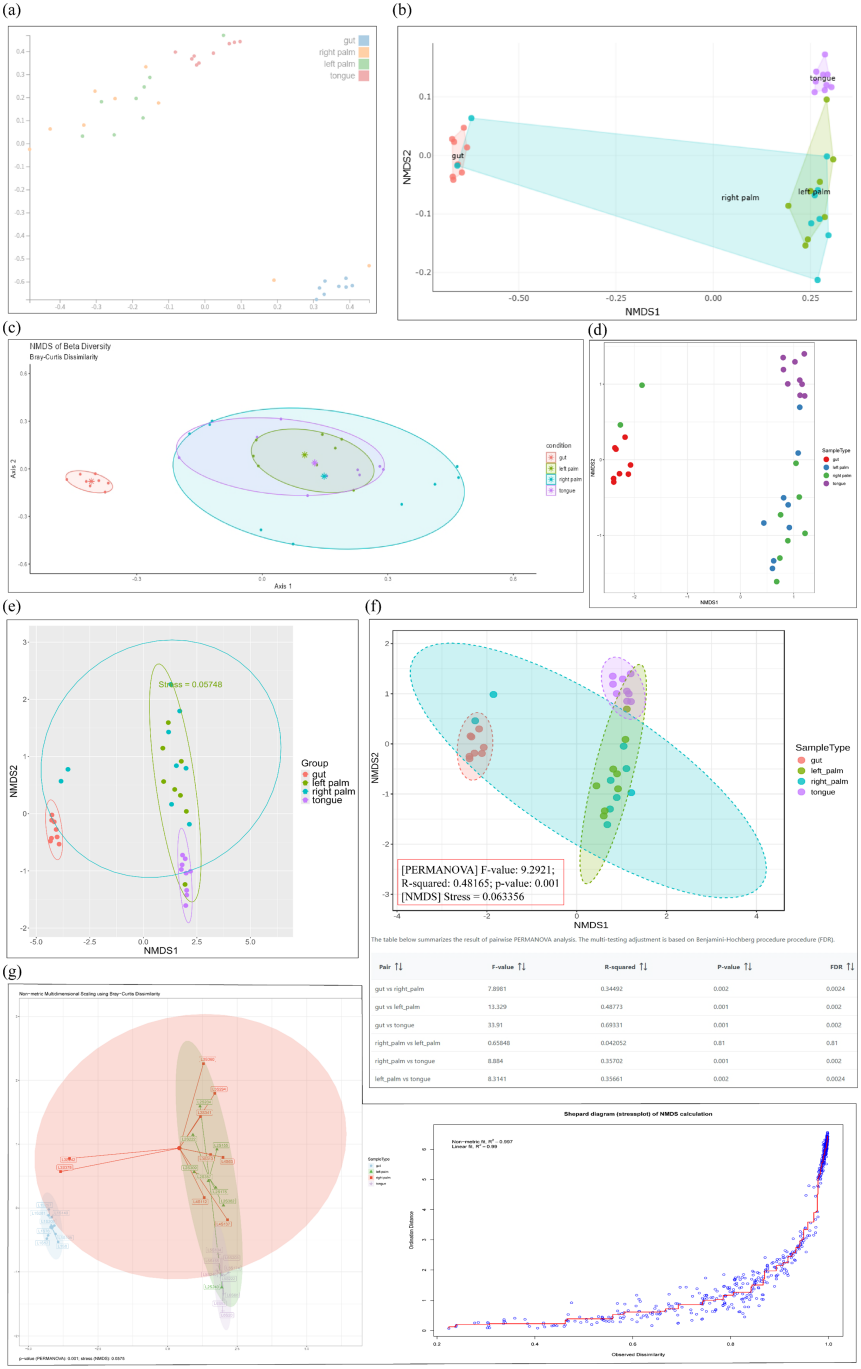
Scatter plot (NMDS) of beta diversity based on the Bray–Curtis distance, generated by **(a)** Mian, **(b)** ampvis2, **(c)** animalcules, **(d)** Shiny-phyloseq, **(e)** wiSDOM, **(f)** MicrobiomeAnalyst 2.0, and **(g)** Namco.

Beta diversity evaluates differences in microbial community composition among samples based on distance metrics, which are subsequently used in ordination analysis. Prior to performing beta diversity analysis, appropriate data transformations are recommended to address the inherent characteristics of microbiome datasets, including zero inflation, compositionality and overdispersion (Lutz et al., 2022). We summarized key features and metrics, encompassing five data visualization approaches, two primary analytical objectives, 16 data transformation strategies, 51 distance metrics (from the *distance* function in the phyloseq package), 11 ordination methods and six statistical tests (Supplementary Table S8). Within beta diversity analysis, the choice of distance metrics and ordination methods is critical for characterizing compositional differences between microbial communities (Cassol et al., 2025). The detailed discussion of how the choice of distance metrics influences the interpretation of microbial community structures can be found in the Fuschi’s report (Fuschi et al., 2025). Moreover, the Bray–Curtis metric is often the most sensitive for detecting group differences, underscoring the need for power analysis and the use of multiple distance metrics (Kers and Saccenti, 2022). Additionally, the applications and comparison of ordination methods was available from review article reported by Armstrong et al. (2022). All tools support scatter plot, each incorporating its own selection of ordination methods. Among these, non-metric multidimensional scaling (NMDS), principal component analysis (PCA), and principal coordinate analysis/multidimensional scaling (PCoA/MDS) are the most widely implemented. Several tools including ampvis2, animalcules, wiSDOM, MicrobiomeAnalyst 2.0, METAGENassist and Namco, also support confidence ellipses in their ordination plots (Figure 6; Supplementary Figure S4). Of these, only wiSDOM, MicrobiomeAnalyst 2.0 and Namco provide statistical tests to evaluate group-level differences, with stress values serving as complementary information to ANOSIM, and only MicrobiomeAnalyst 2.0 implements FDR-based multiple testing correction. Notably, Namco also offers shepard diagram to assess the goodness-of-fit between ordination distances and the original dissimilarity matrix in NMDS ordination space (Tenreiro Machado et al., 2015). Although METAGENassist offers only PCA, it provides a comprehensive set of visualization outputs, including scree plot for component selection, 2D and 3D score plots displaying sample distribution patterns, loading plot indicating key features contributions, and biplot integrating both sample and key feature’s information (Supplementary Figure S4). Additionally, only Mian and MicrobiomeAnalyst 2.0 support complete selection of all taxonomic ranks, whereas the remaining tools offer only partial support or undefined levels.

For heatmap visualization, animalcules employs a sample–sample distance matrix, where both axes represent samples and the cell colors denote pairwise dissimilarity derived from the feature table at the specified taxonomic rank (Supplementary Figure S5a). In contrast, METAGENassist employs a feature–sample abundance matrix in which rows represent taxa and columns correspond to samples, with cell colors reflecting the relative abundance across samples (Supplementary Figure S5b). This approach highlights the differences in community composition and abundance patterns rather than pairwise sample dissimilarities.

For boxplot visualization, two types of boxplots highlight different aspects of community dissimilarity. The first type displays ANOSIM results by partitioning dissimilarity ranks into “within-group” and “between-group” categories, thereby testing whether between-group differences consistently exceed within-group differences. This approach, implemented in animalcules and wiSDOM (Supplementary Figures S6a,b, right), relies on the ANOSIM statistical test and corresponding significance values. The second type visualizes pairwise β-diversity distances within and between groups, providing a quantitative assessment of community divergence across categories. Statistical comparisons (e.g., PERMANOVA or pairwise tests) are frequently superimposed to evaluate significant group-level differences, as illustrated by wiSDOM and Mian in Supplementary Figure S6b (left) and Supplementary Figure S6c, respectively. Notably, animalcules restricts binary group comparisons (Supplementary Figure S6a), whereas wiSDOM and Mian allow multi-group evaluations (Supplementary Figures S6b,c). For dendrogram visualization, Namco, MicrobiomeAnalyst 2.0 and METAGENassist allowed users to intuitively assess hierarchical clustering patterns among samples (Supplementary Figure S7). METAGENassist also supports partitional clustering (e.g., K-means and SOM), which require users to specify the number of clusters (Wang et al., 2005), thereby emphasizing clear cluster assignments (Supplementary Figure S8). Collectively, these visualization strategies demonstrate that beta-diversity analysis can be effectively conducted using either individual visualization methods or their combination. In the module-level evaluation of beta diversity, METAGENassist received the highest score (Figure 4b), largely due to its support for more submodules including heatmaps, dendrograms and SOM, along with its relatively strong performance ratings (Figure 5). Specifically, Mian and MicrobiomeAnalyst 2.0 performed best for scatter plot, while animalcules and METAGENassist demonstrated comparable performance for heatmaps, with METAGENassist, Namco and MicrobiomeAnalyst 2.0 showing similarly strong performance for dendrograms. For box plots, Mian achieved the highest score, and SOM analysis was exclusively supported by METAGENassist.

### 4.5 Taxonomic composition

**Figure 7:**
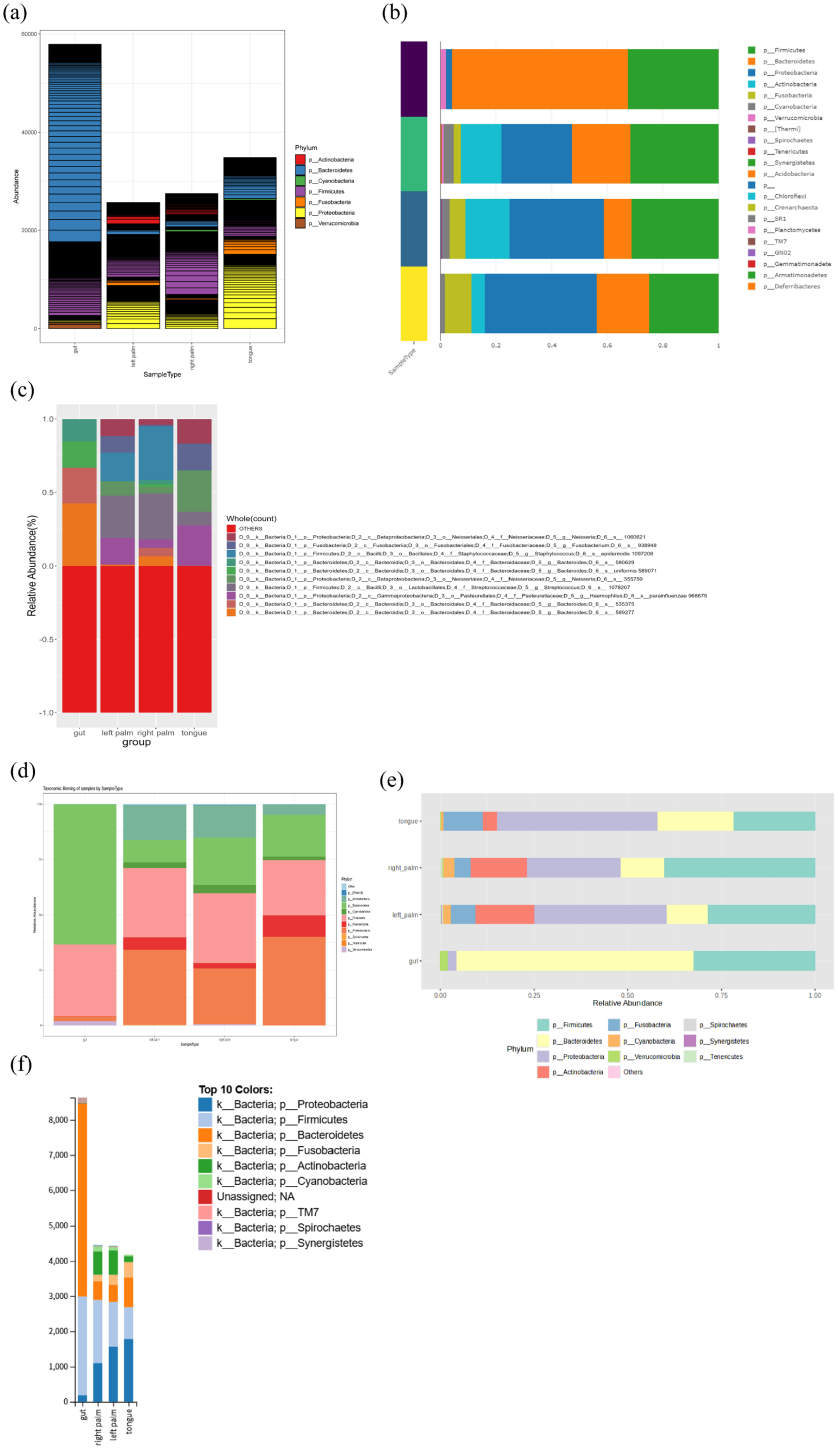
Stacked barplot of phylum-level taxonomic composition produced by **(a)** Shiny-phyloseq, **(b)** animalcules, **(c)** wiSDOM, **(d)** Namco, **(e)** MicrobiomeAnalyst 2.0, and **(f)** Mian.

Taxonomic composition provides an overview of microbial community abundance across hierarchical taxonomic levels in either grouped or individual samples, thereby offering critical insights into community structure and abundance patterns. The visualization methods include stacked bar plot, bar or box plot (for individual taxa), flow or alluvial diagram, sankey diagram, pie or donut chart, heatmaps, heat trees and taxonomic trees. Prior to visualization, the count data are often transformed using relative abundances (Supplementary Table S9). Among available tools, stacked bar plots and heatmaps represent the most widely implemented visualization strategies. We demonstrated the phylum-level taxonomic composition using an example dataset across tools. As shown in the stacked bar plot (Figure 7), the main differences among tools are as follows. With the exception of ampvis2 and METAGENassist, all tools support stacked bar plot. Only Mian enables visualization of abundances across all taxonomic levels, whereas wiSDOM is restricted to feature level. Other tools allow visualization across multiple taxonomic ranks. Moreover, Mian is limited to displaying taxonomic composition based solely on absolute raw counts, while the other tools also accommodate relative abundances. In Shiny-phyloseq, feature-level abundance proportions are represented as areas delineated by black lines within each phylum-level bar. However, features with very low relative abundances tend to be compressed into narrow regions, leading to overly dense boundaries that diminish visual clarity. This constraint may reduce suitability of those plots for publication-quality figures. Additionally, ampvis2, animalcules and METAGENassist support the visualization of individual taxa abundances at various taxonomic levels for individual group or multiple groups (Supplementary Figure S9).

For heatmap visualization (Supplementary Figure S10), Shiny-phyloseq, Namco, Mian and animalcules generally display abundances for individual samples along the x-axis. Notably, animalcules does not display sample labels directly, although both sample and taxa names can be retrieved interactively by cursor over. In contrast, ampvis2 and MicrobiomeAnalyst 2.0 support abundances visualization at the both individual sample and group levels. Furthermore, Shiny-phyloseq and Namco allow samples to be ordered by ordination results along the x-axis while displaying taxon abundances on the y-axis, enabling simultaneous visualization of community composition and sample similarity. MicrobiomeAnalyst 2.0, METAGENassist and Mian also offer pie and donut charts (Supplementary Figure S11). However, Mian displays only the top 10 taxa, and full taxon names are available only via mouse-hover interactions, which hinders the production of publication-quality figures and requires additional manual annotation.

Finally, the tree plot in MicrobiomeAnalyst 2.0 (heat tree, Supplementary Figure S12a) and Mian (taxonomic tree, Supplementary Figure S12b) offers more informative visualization than traditional stacked bar or pie charts, which overlook the hierarchical nature of taxonomic classifications and are restricted by color limitations that restrict the number of taxa that can be effectively displayed (Foster et al., 2017). By leveraging the taxonomic hierarchy, heat tree plot by MicrobiomeAnalyst 2.0 quantitatively illustrates abundance profiles using median abundance values (Supplementary Figure S12a). These heat trees are valuable for assessing taxonomic coverage, identifying barcode biases and visualizing differences in taxon abundance among communities (Foster et al., 2017). In Supplementary Figure S12b, the taxonomic tree plot by Mian facilitates comparison of taxon abundances across taxonomic level among groups. For the evaluation of taxonomic composition, Mian and MicrobiomeAnalyst 2.0 ranked the highest overall (Figure 4c), demonstrating strong performance across all submodules. However, both tools lack support for bar/box plots, which are well implemented in ampvis2, animalcules and METAGENassist (Figure 5).

### 4.6 Differential comparison

**Figure 8:**
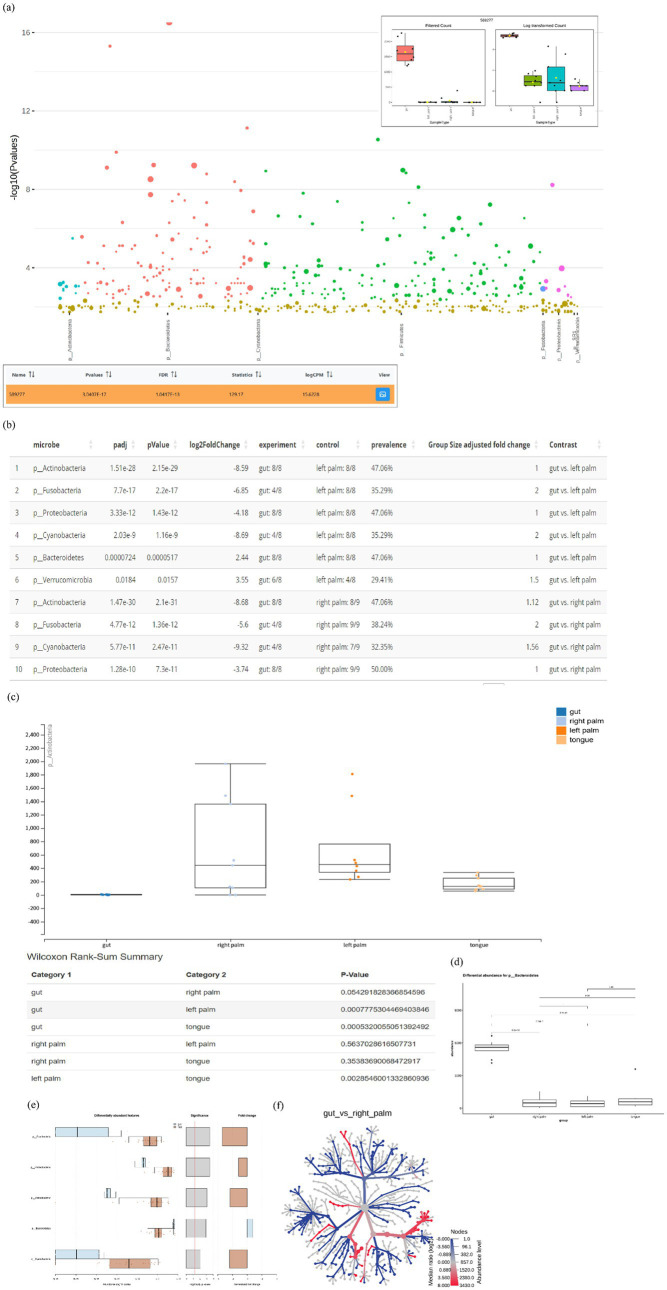
Visualization of differential comparisons of taxa: **(a)** Manhattan plot from MicrobiomeAnalyst 2.0; **(b)** tabular output from animalcules; **(c)** extended bar/box plot from Mian; **(d)** extended bar/box plot from Namco; **(e)** association plot from Namco; and **(f)** heat tree from MicrobiomeAnalyst 2.0.

Differential comparison is to identify features, such as taxa, functionality or gene expression patterns, that exhibit statistically significant differences across experimental groups or conditions. As summarized in Supplementary Table S10, seven visualization approaches and 12 statistical methods for differential analysis were cataloged. Different tools employ distinct visualization and statistical strategies to represent differences in microbial abundance. For example, animalcules support only tabular outputs, whereas Mian and Namco also provide boxplots. Notably, MicrobiomeAnalyst 2.0 provides heat trees and Manhattan plots, which are interactive with boxplots and tabular outputs (Figure 8a–d). The association plot implemented in Namco, which optionally incorporates effect size metrics such as fold-change, AUC and prevalence shift, is restricted to pairing comparisons (Figure 8e). The heat trees are used for differential analysis and taxonomic composition in MicrobiomeAnalyst 2.0. For differential analysis, the heat tree displays pairwise abundance differences using color gradients that encode significant shifts in median ratio (log_2_). In Figure 8f, taxa highlighted in red indicate enrichment in the gut, whereas dark blue indicates enrichment in the right palm. METAGENassist also provides volcano and manhattan plots to visualize statistical comparisons of microbial features across groups (Supplementary Figure S13). The volcano plot supports pairing comparisons by showing both the magnitude of change (log₂ fold change) and statistical significance (−log₁₀ *p*-value) for each feature (Supplementary Figure S13a), whereas the manhattan plot is suited for multi-group comparisons (Supplementary Figure S13b). Moreover, tools offering multiple testing correction include MicrobiomeAnalyst 2.0 (FDR), METAGENassist (FDR), animalcules (adjusted *p*-value), Mian (FDR) and Namco (FDR or FWER). Actually, the number and identity of significant features for differential abundance analysis depend on the chosen statistical test and data preprocessing, as well as on sample size, sequencing depth and the effect size of community differences (Nearing et al., 2022). For the evaluation of differential comparison, nearly all submodules were supported by one or two tools (Figure 5). MicrobiomeAnalyst 2.0 received the highest score (Figure 4d), primarily due to its strong performance in two submodules (Figure 5). Researchers may therefore select the most appropriate submodule based on their analytical preferences.

### 4.7 Network analysis

Network analysis is widely applied to infer potential interactions among microbial taxa, thereby elucidating mutualistic, commensal, parasitic or competitive relationships. As summarized in Supplementary Table S11, network construction can be performed either at the sample level (supported by Mian and Shiny-phyloseq) or taxonomic levels. Across tools, networks are generated using 46 distance-based or seven correlation-based algorithms, with visualization commonly rendered through 14 distinct layout strategies. An overview of approaches from correlation-based approaches to complex conditional dependence methods has been conducted, along with a discussion of current limitations in network analysis (Matchado et al., 2021). Furthermore, network characterization typically involves quantifying topological attributes, including node degree, betweenness centrality and modularity, to identify keystone taxa and functionally coherent community modules. Key differences across tools are summarized in Supplementary Table S11. Firstly, all four platforms support the construction of co-occurrence networks using feature tables aggregated to predefined taxonomic levels. Shiny-phyloseq only limit to the feature level, whereas Mian enables network inference across all taxonomic levels. Secondly, Namco and Mian rely on correlation coefficients, Shiny-phyloseq exclusively adopts distance-based similarity measures, and MicrobiomeAnalyst 2.0 integrates both correlation- and distance-based approaches. Thirdly, although Shiny-phyloseq provides the most diverse set of layout options for network visualization, neither MicrobiomeAnalyst 2.0 nor Mian offers customizable layout configurations.

We additionally compared the network visualization across the tools (Supplementary Figure S14). Shiny-phyloseq does not provide taxonomic abundance information and node labels, although it supports color-coded annotations at higher taxonomic ranks. It also incorporates 3DNetwork functionalities, facilitating interactive exploration through dynamic 3D animations. Mian provided limited visualization of relative abundances, taxonomic levels and correlation strengths, with node labels accessible only upon hovering. MicrobiomeAnalyst 2.0 displays node labels and abundances information but does not encode correlation strength through edge thickness. In contrast, Namco offers the most comprehensive visualization features, including explicit node labels, edges thickness corresponding to correlation strength, hub identification via eigenvector centrality and systematic reporting of network topological properties, making it the robust platform for co-occurrence network analysis. For the evaluation of network analysis, Namco demonstrated the strongest overall performance, followed by Shiny-phyloseq, MicrobiomeAnalyst 2.0 and Mian (Figures 4e, 5).

### 4.8 Correlation analysis

Correlation analysis is to reveal significant associations either among taxa or between taxa and continuous experimental variables (i.e., alpha diversity), which are visualized through scatter plots (Mian), heatmaps (Mian, METAGENassist and Namco) or bar plots (MicrobiomeAnalyst 2.0) (Supplementary Table S12; Supplementary Figure S15). In Mian, scatter plots depict relationships between two variables (e.g., taxonomic abundances or environmental factors) along the x- and y-axes, accompanied by statistical tests that provide both the correlation coefficient and the associated *p*-value. Namco also utilize heatmaps to depict pairwise correlations between taxa and associated variables (e.g., taxa versus alpha diversity), whereas MicrobiomeAnalyst 2.0 employs bar plots to represent one-to-many correlation patterns (e.g., OTU1 correlated with OTU2, OTU3, and OTU4), integrating abundance information across sample groups and FDR-adjusted significance. It is worthing noting that the choice of correlation methods is critical for accurately characterizing relationships among variables in microbiome studies. The performance of eight correlation techniques for microbial correlation analysis has been benchmarked, highlighting their respective strengths and limitations, and providing recommendations for future research and toolkit application (Weiss et al., 2016). For the evaluation of correlation analysis, Mian achieved the highest overall score among all assessed tools (Figure 4f), with particularly strong performance in heatmap and scatter-plot visualizations (Figure 5).

### 4.9 Functional analysis

Functional prediction in amplicon-based microbiome studies aims to infer the metabolic potential and ecological roles of microbial communities. Commonly used tools include PICRUSt, PICRUSt2, Tax4Fun, Tax4Fun2, BugBase and FAPROTAX for bacterial communities, as well as FunGuild and FunFun for fungal communities, which enable estimation of functional gene abundances, pathway coverage and ecological traits (Supplementary Table S13). A total of 24 functional inference tools were systematically summarized, covering their implementation, targeted genes, functional prediction capabilities, underlying approaches, analytical methods, required input data, strengths and specific features, and known limitations (Djemiel et al., 2022). These predicted functions can be further integrated with statistical analysis and diverse visualization approaches to assess functional differences across experimental conditions. Among those tools, only Namco and MicrobiomeAnalyst 2.0 support bacterial functional prediction. In contrast, wiSDOM was unable to perform this analysis reliably due to the absence of the embedded R package “themetagenomics.” Functional prediction was conducted using the example datasets provided by the tools, as our test dataset (Caporaso dataset in the *phyloseq* package) lacks the required RefSeq data. Namco provides a more extensive suite of functional outputs, including boxplots and volcano plots with FDR-adjusted pairwise comparisons, whereas MicrobiomeAnalyst 2.0 offers boxplots and tabular summaries of functional abundance without additional statistical testing (Supplementary Figure S16). For the evaluation of functional analysis, MicrobiomeAnalyst 2.0 scored slightly higher than Namco (Figure 4g), primarily due to its support for multiple submodules (Figure 5), despite Namco offering more advanced statistical analysis methods.

### 4.10 Machine learning

Machine learning (ML) applied to large-scale microbiome datasets serves three principal objectives: (1) microbial classification and taxonomic assignment; (2) prediction of host phenotypes by linking microbial populations to phenotypic and environmental traits; and (3) elucidation of host–microbiome interactions and applications (e.g., biomarker discovery) (Marcos-Zambrano et al., 2021). According to the review by Marcos-Zambrano et al., ML approaches encompass 16 algorithms categorized into five methodological groups (Supplementary Table S14). Biomarker analysis is commonly performed using logistic regression, LEfSe algorithm and random forests approaches. The tools wiSDOM and Namco were excluded from detail evaluation, because their visualization outputs may be unreliable, due to web interface limitations in wiSDOM and prolonged non-responsiveness in Namco. The animalcules enables biomarker identification via logistic regression and random forest classification models, with predictive performance evaluated using AUC metrics and averaged cross-validated ROC curves. MicrobiomeAnalyst 2.0 provides graphical summaries of classification results based on LEfSe and random forest algorithms, along with abundance distributions of identified biomarkers across sample groups (Supplementary Figure S17). In Mian, ML is applied to evaluate the performance of linear models and random forest classifiers in predicting continuous experimental variables (linear regressor) and categorical variables (linear classifier and random forest classifier), as well as to evaluate the predictive capacity of deep neural networks for both categorical or numerical outcomes. METAGENassist also provides useful information such as OOB (out-of-bag) error, feature importance, outlier detection through random forest analysis (Supplementary Figure S18), and performs support vector machines (SVM) analysis (Supplementary Figure S19). For the evaluation of machine learning, animalcules, MicrobiomeAnalyst 2.0 and METAGENassist achieved the highest and identical scores (Figures 4h, 5), indicating comparable capabilities in implementing machine-learning-based analysis.

### 4.11 Tree plot

The tree plot is a hierarchical visualization approach used to depict the taxonomic structure of microbial communities. In these visualizations, nodes typically represent taxa, whereas branch lengths and topological arrangements reflect phylogenetic distances or taxonomic ranks. Additional attributes, such as relative abundance or prevalence, may be encoded through variations in node size, color gradients or annotated labels. As illustrated in Supplementary Figure S20, Shiny-phyloseq, Namco and MicrobiomeAnalyst 2.0 all support the construction of taxonomic trees. In Shiny-phyloseq and MicrobiomeAnalyst 2.0, nodes are annotated with symbolic markers or colored blocks to indicate taxon presence, whereas MicrobiomeAnalyst 2.0 additionally scales node size according to taxon abundance across samples. In contrast, Namco displays features annotated at higher taxonomic ranks, showing either count occurrences or mean abundances across groups. Notably, MicrobiomeAnalyst 2.0 support higher taxonomic resolution, while Shiny-phyloseq and Namco restrict node representation to the feature level. For the evaluation of tree-plot, animalcules and MicrobiomeAnalyst 2.0 outperformed Namco and Shiny-phyloseq (Figures 4i, 5).

### 4.12 User experience

All evaluated tools provide the ability to download high-resolution figures suitable for publication. Nevertheless, the accessibility of certain web-based platforms can be inconsistent due to intermittent service disruptions. For example, MicrobiomeAnalyst 2.0, Namco, ampvis2, and METAGENassist were occasionally unresponsive following periods of inactivity or network congestion, requiring data re-upload and thereby prolonging analysis time. In contrast, tools developed using the R shiny framework, such as animalcules, Shiny-phyloseq and wisdom, as well as Mian demonstrated stable performance and consistent accessibility. Furthermore, several tools, including Mian, Namco, animalcules and ampvis2, offer interactive visualizations, enabling researchers to enhance usability and analytical flexibility. For the evaluation of user experience, animalcules and Mian achieved the highest scores, indicating superior usability compared to the other tools (Figures 4j, 5). Overall, MicrobiomeAnalyst 2.0 achieved the highest score among the evaluated tools (Figure 4k), aligning with its status as one of the most influential platforms based on total citations and average annual citations. Mian and Namco ranked second and third, respectively, despite lower citation counts, likely due to their recent publication in 2022 and limited exposure within the research community.

### 4.13 Other modules

Additional analysis that are not commonly used will be described in the following sections. (1) In Mian, feature selection analysis was applied to (i) identify discriminative features or taxonomic groups between sample groups (Boruta classification, Elastic Net classification, Fisher’s exact test and differential selection), (ii) determine features predictive of quantitative metadata variables (Elastic Net regression), and (iii) detect features that exhibit significant correlations with other microbial, functional or diversity-related variables (correlation-based selection). (2) In Namco, the co-occurrence, network inference and differential network modules collectively enable the identification of taxa co-occurrence patterns, the construction of feature-based microbial networks, and the comparative analysis of network structures across sample groups. Topic modeling is used to identify features with similar associations or behavioral patterns, while time-series analysis captures temporal changes in taxa dynamics. We also strongly recommend the recently published ggClusterNet, which enables comprehensive microbial co-occurrence network analysis and visualization. It provides a robust, efficient and user-friendly framework that supports reproducible workflows and offers versatile visual outputs for exploring co-occurrence patterns and indicator species correlations (Wen et al., 2025). Other functions in Namco, such as confounding analysis and multi-omics analysis, represent advanced functionalities with the potential to become research hotspots in the future. (3) In MicrobiomeAnalyst 2.0, core microbiome analysis use barplot to depict the set of taxa that are detected in a high fraction of the population above a given abundance threshold of relative abundance and sample prevalence. The multiple linear regression module employs generalized linear models to identify associations between microbial features and experimental metadata. (4) Additionally, METAGENassist employed Partial Least Squares Discriminant Analysis (PLS-DA) as a supervised method to visualize group separation, with cross-validation/permutation testing used to validate the PLS-DA results and mitigate the risk of overfitting. METAGENassist also supports phenotype-based analysis to derive phenotypic traits from taxonomic input data across nearly 20 functional categories, including GC content, genome size, oxygen requirements, energy sources and preferred temperature range. Users can use this phenotypically enriched dataset to perform a range of univariate and multivariate analysis, such as fold change analysis, t-tests, PCA, PLS-DA, clustering and classification, enabling comprehensive exploration of functional patterns and differences among samples.

## 5 Limitations, suggestions and prospects of web-based tools for microbiome analysis

### 5.1 Limitations

The limitations of existing tools can be summarized as follows. Firstly, the accessibility of some web-based tools may be unstable, particularly when they remain inactive for an extended period. In contrast, locally deployed R Shiny applications generally offer superior stability and reliability than browser-based web tools, with fewer errors arising from internet connectivity or server-side limitations. Secondly, the diversity of input data formats required across tools limited interoperability and imposes additional burdens for beginners. Thus, the standardization of input data formats across platforms is desirable, even though the detail instructions are provided by most tools. Thirdly, the limited range of selectable features or options in some tools constrains their applicability. Therefore, incorporating additional features, such as statistical methods and distance metrics, would provide users with greater flexibility in choosing options suited to their datasets. Fourthly, although some tools offer interactive visualization that displays key information (e.g., sample names or taxa names), these elements are often absent in the exported figures, which reduces their utility for publication.

### 5.2 Suggestions

Some functionalities are not natively implemented in some tools but can be achieved through external preprocessing. For example, ampvis2 does not provide built-in data normalization, thereby requiring users to reformat and normalizing their data in advance using other tools or the R environment. Recently, ImageGP 2 has been introduced as an advanced platform for enhanced data visualization and reproducible analysis in microbiome research, offering high-quality figure generation along with integrated scripting capabilities (Chen et al., 2024). Moreover, graphical user interfaces may be used for preliminary exploration for coding-proficient users, followed by command-line functions and R markdown scripting to produce reproducible reports. For example, EasyAmplicon is a user-friendly pipeline providing a streamlined workflow from raw data to results with a detailed protocol and video guide, enabling researchers to perform all analysis without advanced scripting skills (Yousuf et al., 2024). Lastly, interactive visualization and figure export functionalities within tools should be more seamlessly integrated. Specifically, users should be able to define the key information displayed during interactive exploration, and the information that user required should be consistently preserved in the exported figures.

### 5.3 Prospects

The future prospects of web-based tools for microbiome analysis can be summarized as follows. Firstly, more advanced functional modules, such as comprehensive functional profiling, enhanced data visualization methods, robust significance testing algorithms, and improved data filtering and transformation methods, may be fully supported. Secondly, integrated multi-omics analysis including metagenome, metabolomics and transcriptomics etc., will become increasingly important for elucidating microbial functions and their ecological interactions. For example, the Wekemo Bioincloud platform provides metabolomics, proteomics, genomics and multi-omics association analysis, equipped with 22 workflows and 65 visualization tools, making it a user-friendly and widely adopted solution for processing diverse datasets (Gao et al., 2024). Thirdly, meta-analysis leveraging compatible public datasets may facilitate the incorporation of contextual references and enable broader pattern discovery.

## 6 Conclusion

Amplicon sequencing provides rapid and cost-effective approaches for microbiome profiling, with a variety of R-based and web-based tools for subsequent analysis. In this review, we comprehensively compared metrics available across modules (alpha and beta diversity, taxonomic composition, differential comparison, network and correlation analysis, functional profiling, machine learning, tree-plot and user experience) and conducted performance comparison in eight web-based tools that are freely accessible and easily used for beginners without scripting knowledge and bioinformatics training. All tools share less data filtering options and normalization methods. Our results reveal that performance varies considerably across tools and analytical modules. Specifically, Mian and MicrobiomeAnalyst 2.0 excelled in alpha diversity analysis, particularly in generating rarefaction curves and box plots, while METAGENassist demonstrated superior performance in beta diversity analysis due to its broader support for submodules such as heatmaps, dendrograms and SOM. In taxonomic composition analysis, Mian and MicrobiomeAnalyst 2.0 ranked highest overall, although bar and box plot visualizations were better implemented in ampvis2, animalcules and METAGENassist. For differential comparison, MicrobiomeAnalyst 2.0 scored highest but supports only two submodules, emphasizing careful selection based on analytical objectives. Network analysis was most effectively supported by Namco, whereas correlation analysis performance was strongest in Mian, particularly for heatmaps and scatter plots. Functional analysis favored MicrobiomeAnalyst 2.0, despite Namco providing more advanced statistical methods. Machine-learning-based analysis were comparably implemented in animalcules, MicrobiomeAnalyst 2.0 and METAGENassist, while treeplot visualization was most effective in animalcules and MicrobiomeAnalyst 2.0. For user experience, animalcules and Mian received the highest evaluation scores. Overall, MicrobiomeAnalyst 2.0 achieved the highest cumulative performance, consistent with its prominence as a highly cited platform, whereas Mian and Namco ranked second and third, likely reflecting their more recent introduction to the field. Web-based tools for microbiome analysis also have several limitations remain. Accessibility and stability can be inconsistent, particularly for browser-based platforms, while diversity in input data formats and limited selectable features restrict interoperability and analytical flexibility. Interactive visualizations often fail to retain key information in exported figures, reducing their utility for publication. To address these challenges, functionalities not natively implemented should be supported through preprocessing or integrated into tool workflows, and interactive visualization should be seamlessly combined with figure export to preserve user-defined information. Advanced features such as robust statistical methods, enhanced data visualization and multi-omics integration will further enhance the applicability of these tools. Moreover, meta-analytical capabilities using public datasets can facilitate broader pattern discovery and contextual interpretation. Collectively, these improvements will increase the reliability, flexibility and interpretability of web-based microbiome analysis platforms, supporting more comprehensive and reproducible research outcomes. Our findings provide a comprehensive reference for researchers in selecting the most suitable web-based tools for specific microbiome analysis tasks, emphasizing the importance of considering both module-specific performance and tool-specific capabilities.
