# Supplementary material for: A comprehensive comparison of web-based tools for amplicon-metagenomic analysis
Source: Front Microbiol. 2026 Jan 6;16:1711000. doi: 10.3389/fmicb.2025.1711000 (PMC12815881; doi:10.3389/fmicb.2025.1711000)
Supplement: Supplementary file 1 [file Supplementary_file_1.zip › Supplementary Figures S1-S20.DOCX]

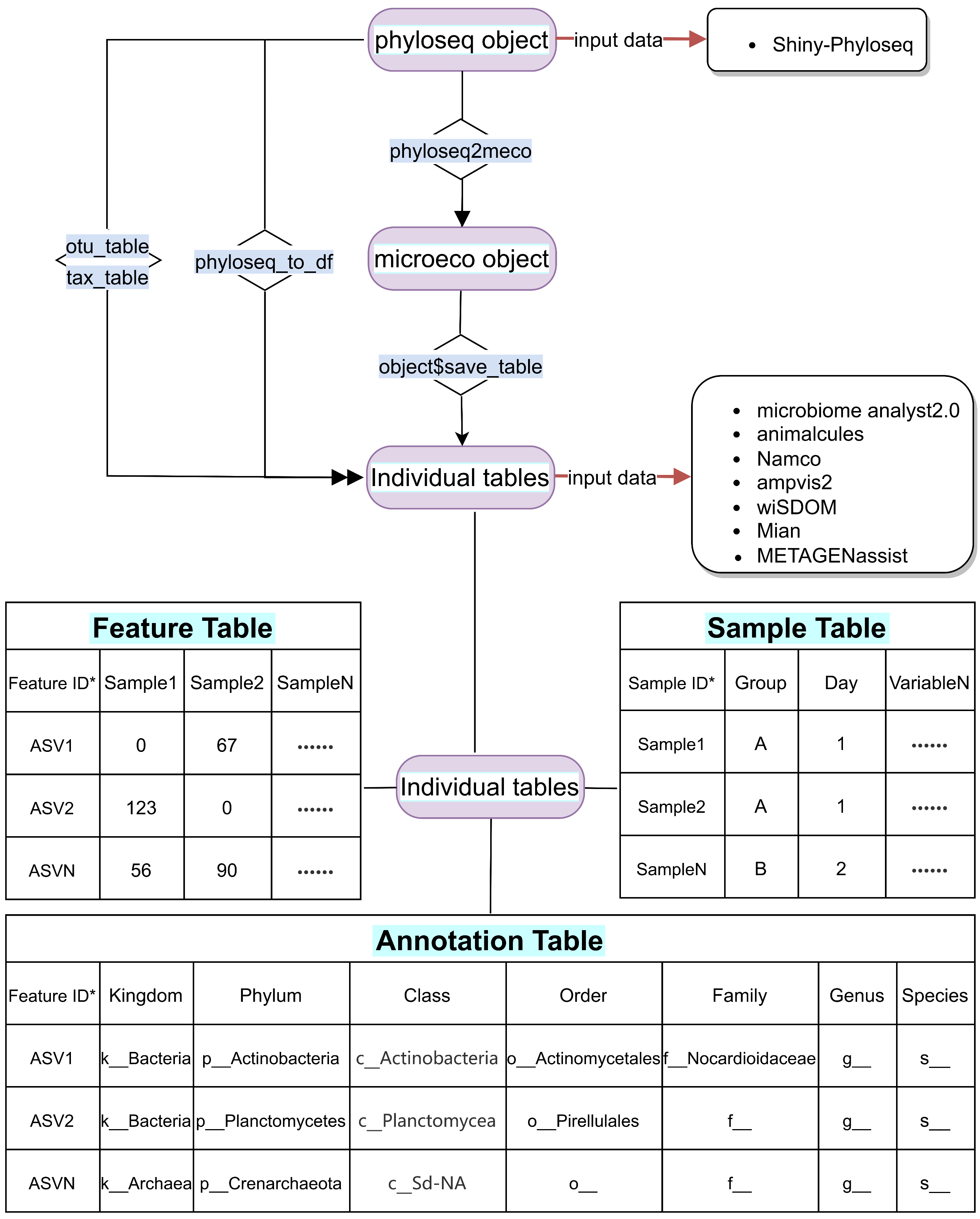


**FIGURE S1** Workflow for generating individual input data files from phyloseq object.


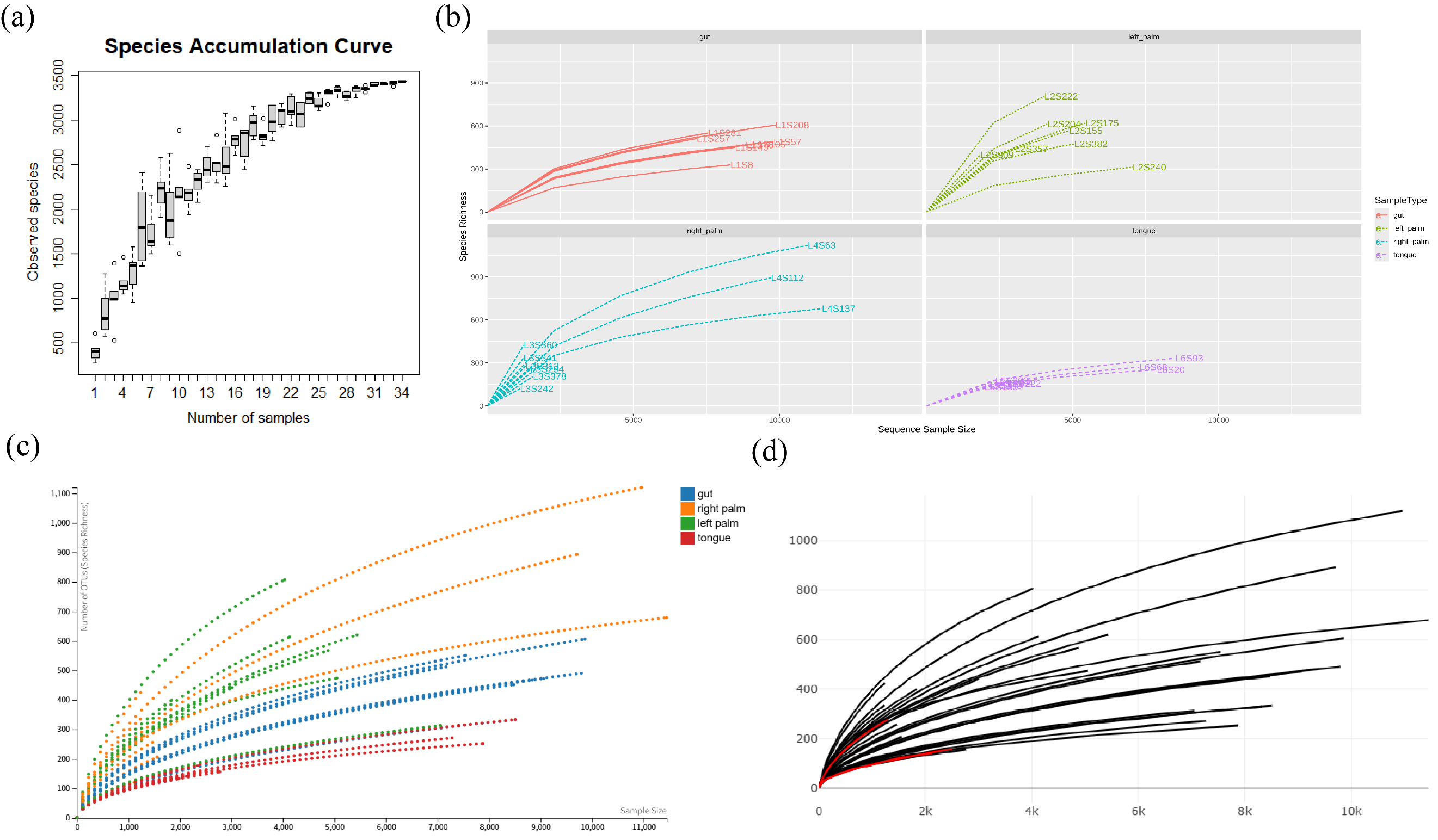


**FIGURE S2** Rarefaction curves generated by tools of (a) wiSDOM, (b) MicrobiomeAnalyst 2.0, (c) Mian and (d) Namco.


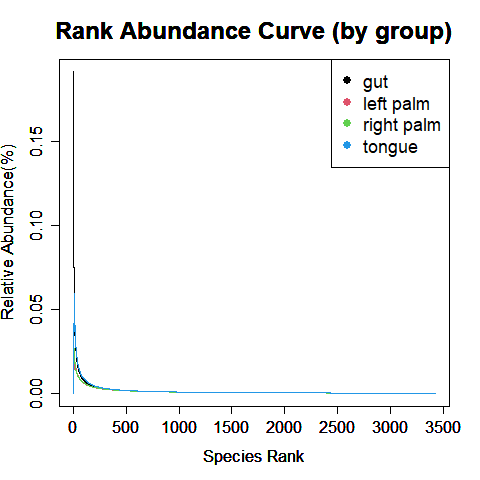


**FIGURE S3** The rank abundance curve was plot by wiSDOM.


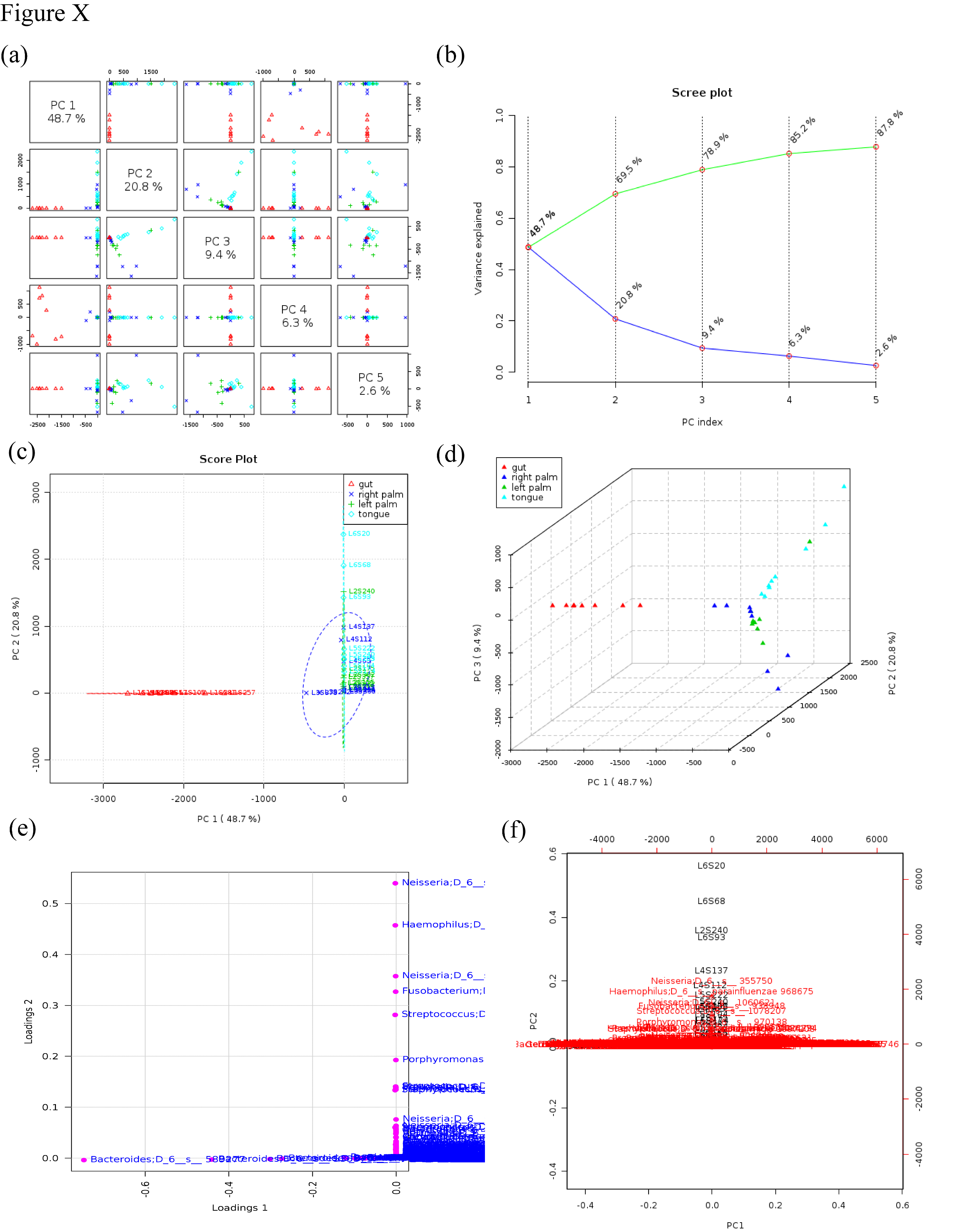


**FIGURE S4** PCA analysis performed using METAGENassist. (a) overview plot, (b) scree plot, (c) 2D score plot, (d) 3D score plot, (e) loading plot and (f) biplot.


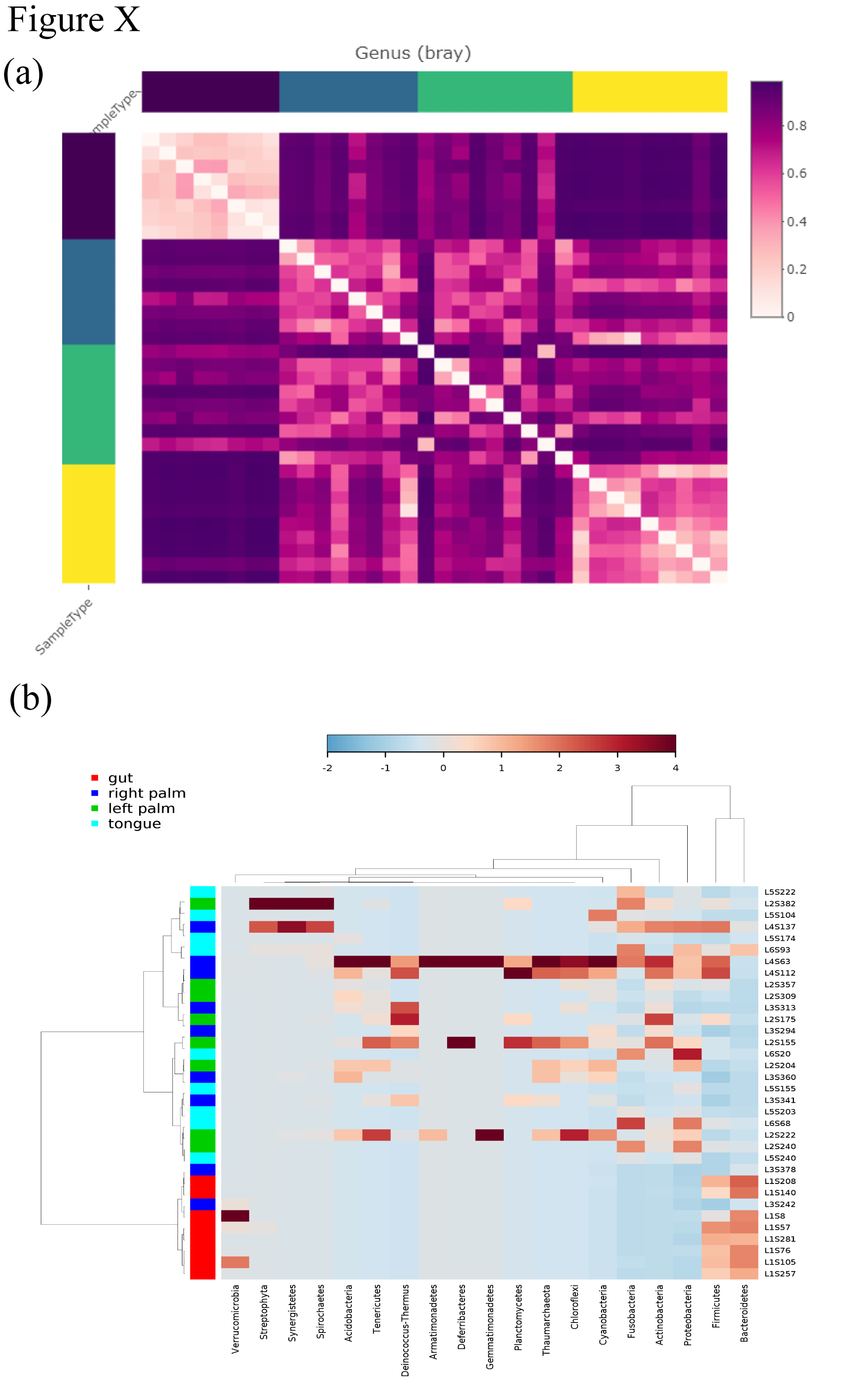


**FIGURE S5** Heatmap plot of beta diversity produced by (a) animalcules and (b) METAGENassist.


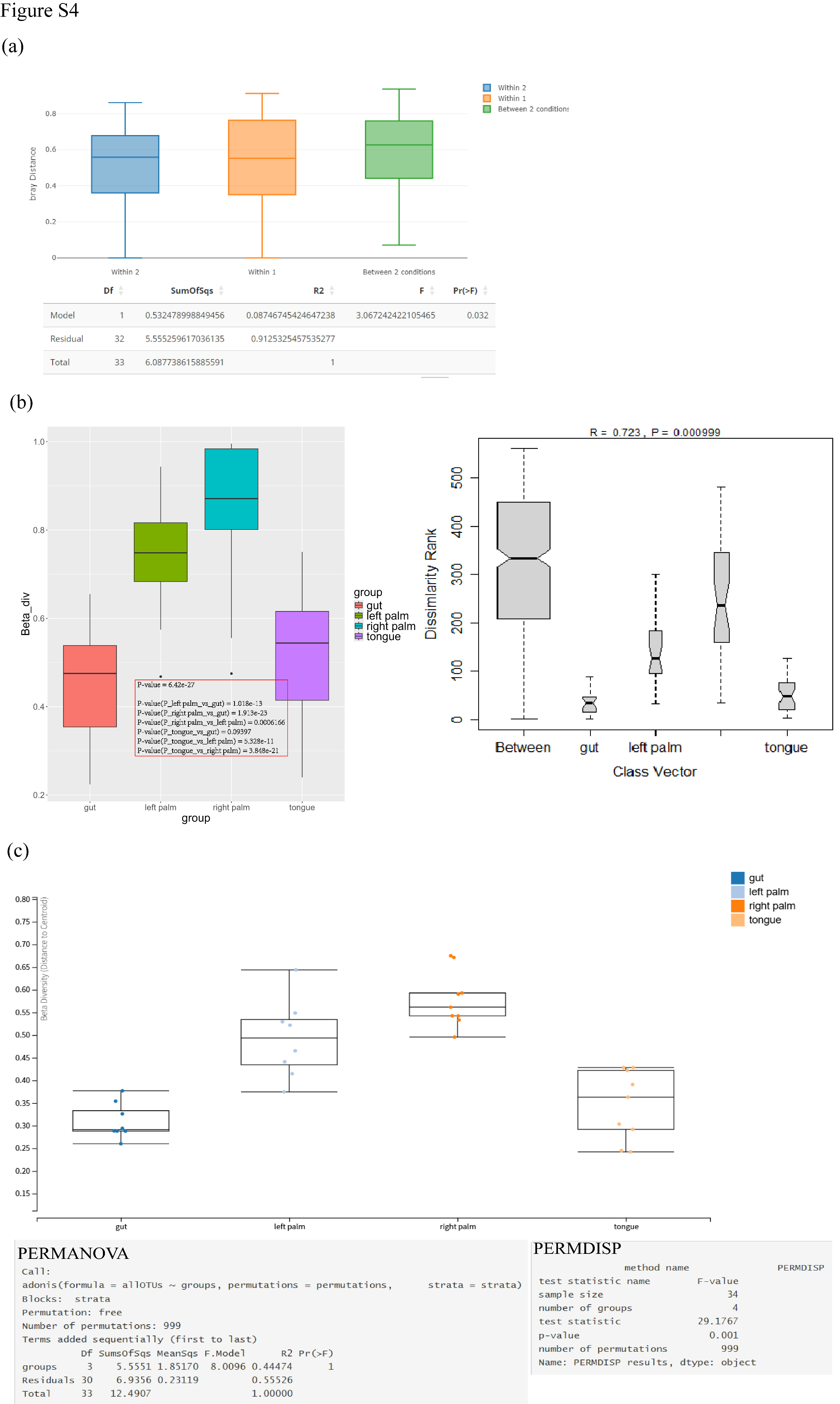


**FIGURE S6** Boxplot plot of beta diversity produced by (a) animalcules, (b) wiSDOM and (c) Mian.


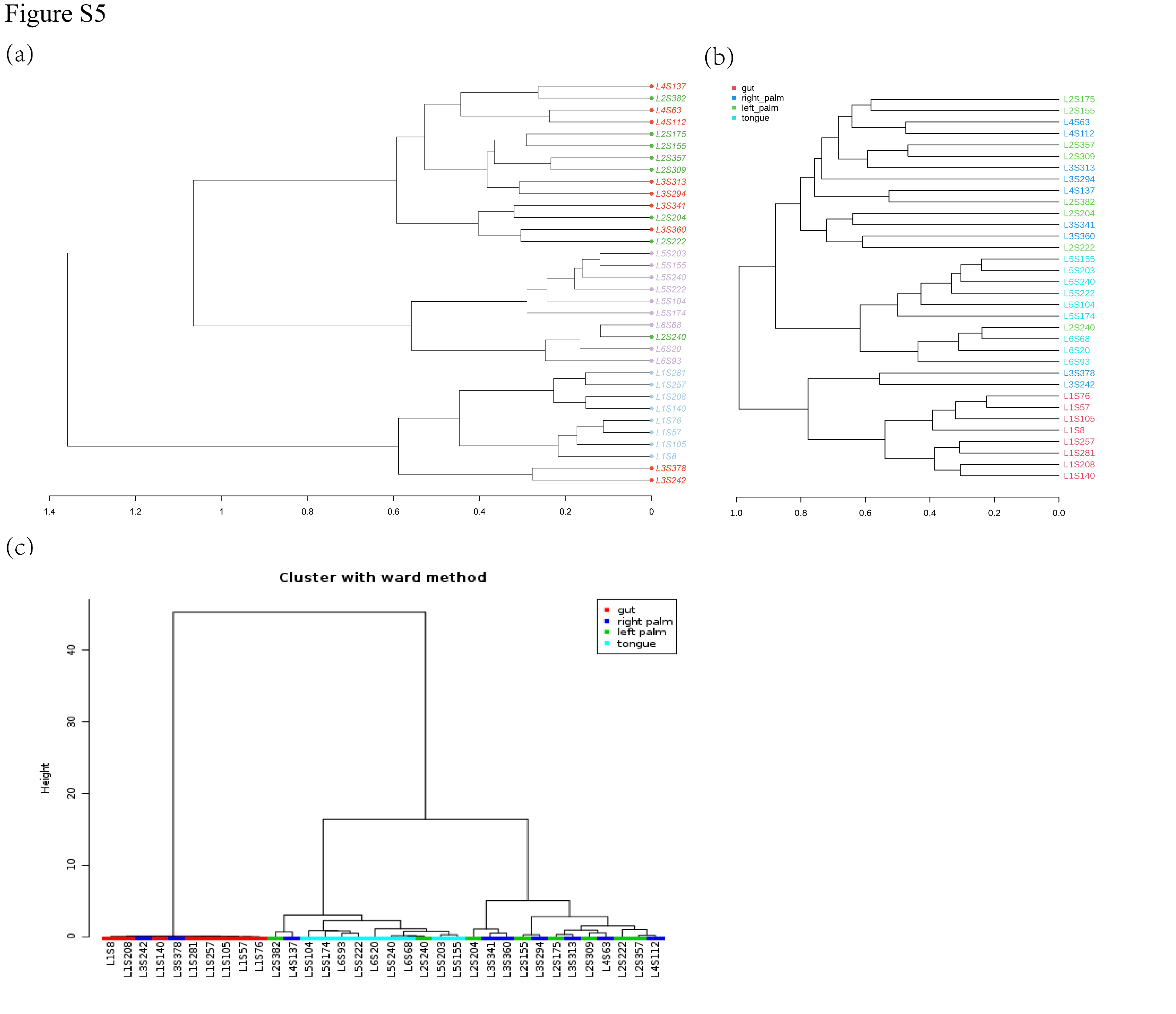


**FIGURE S7** Dendrogram showing the sample clustering using (a) Namco, (b) MicrobiomeAnalyst 2.0 and (c) METAGENassist.


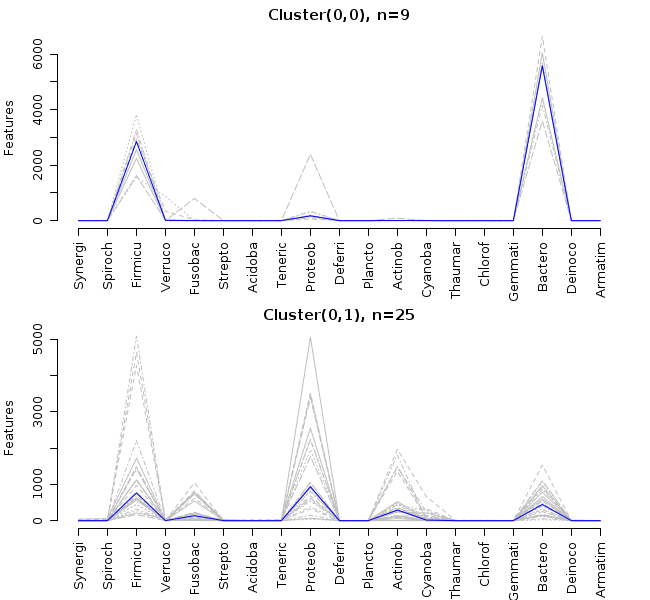


**FIGURE S8** Partitional clustering performed using METAGENassist.


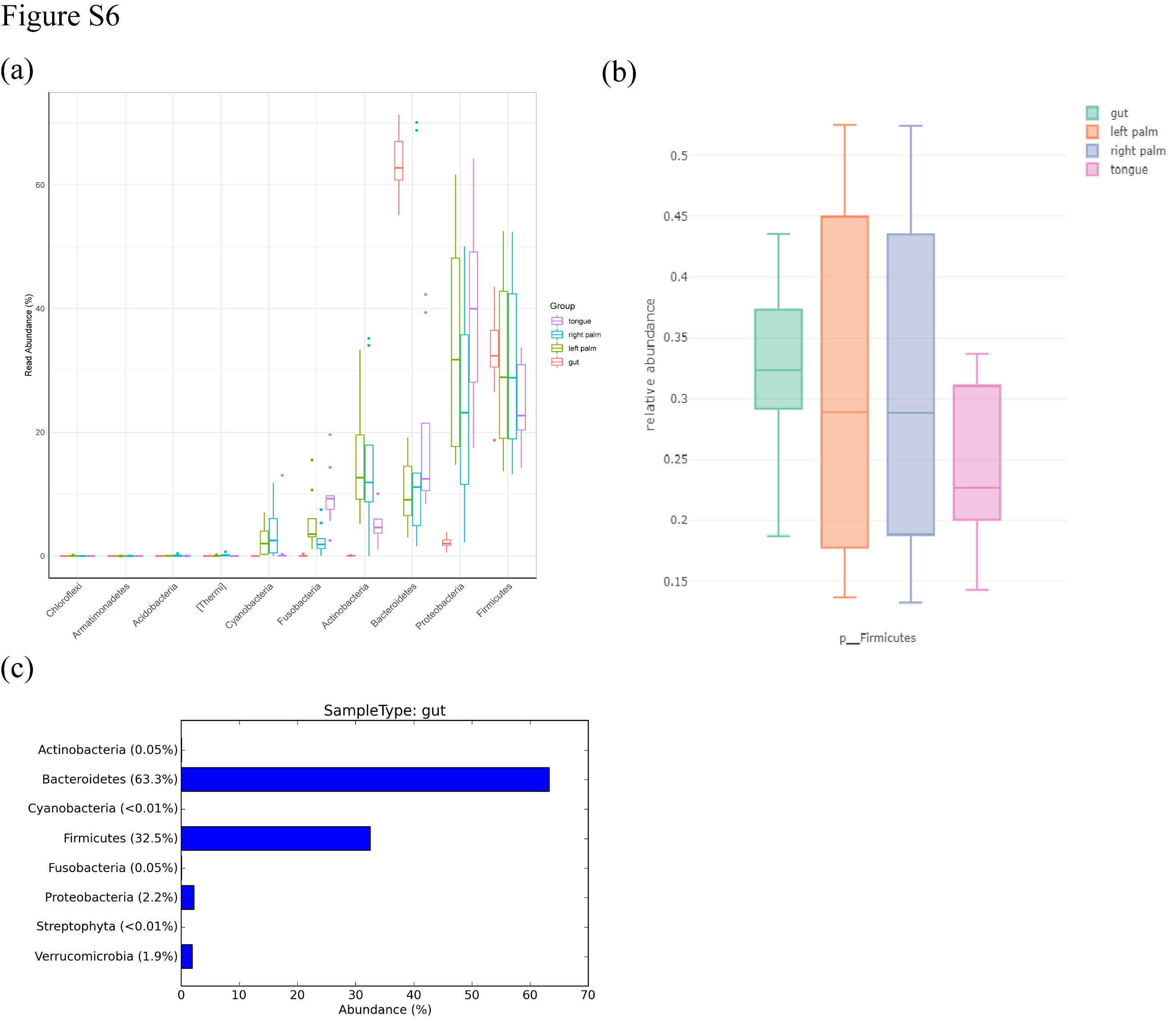


**FIGURE S9** Barplots or Boxplots of microbial taxa at phylum level were generated by (a) ampvis2, (b) animalcules and (c) METAGENassist.


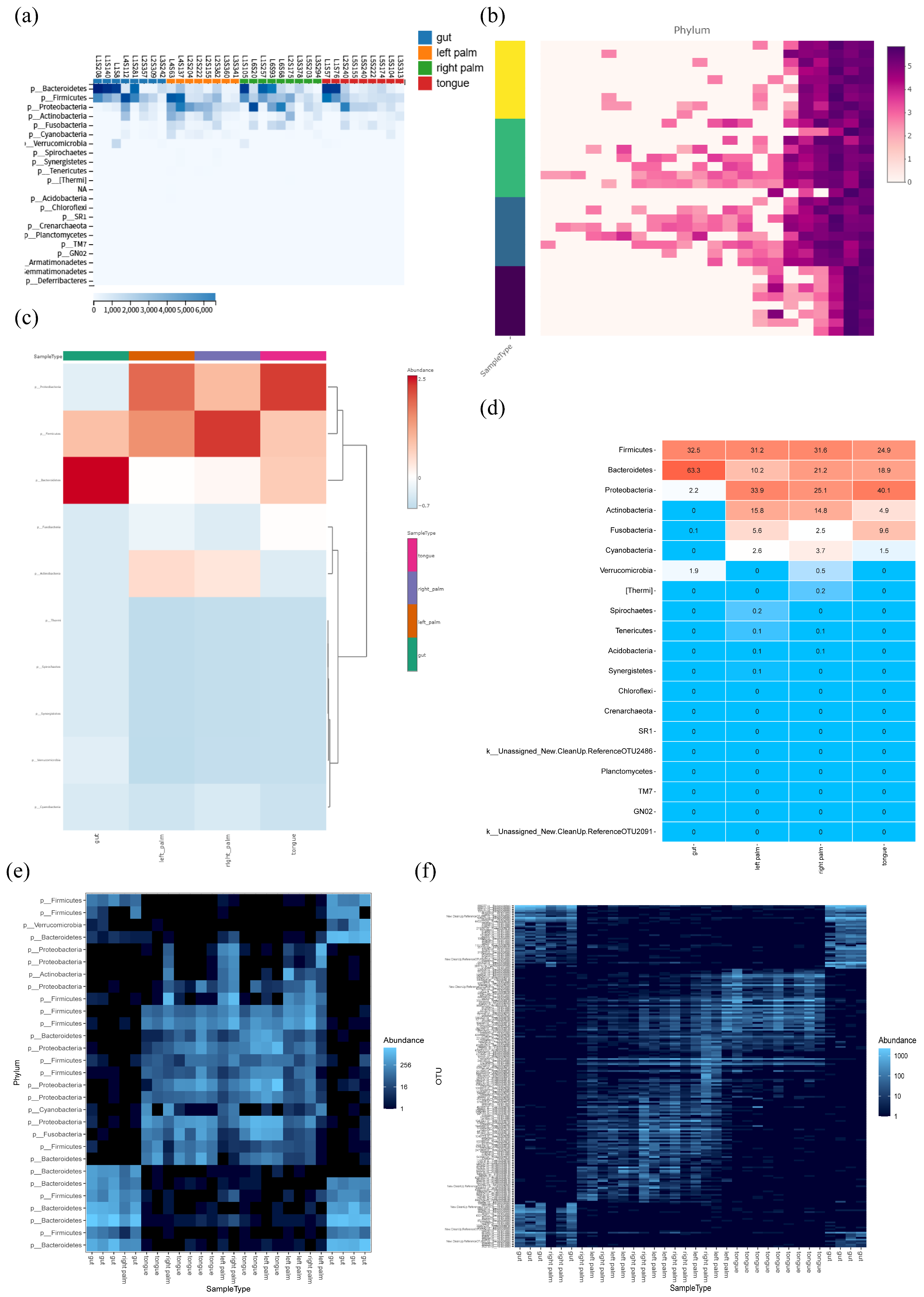


**FIGURE S10** Heatmap of microbial taxa at phylum level were generated using the example dataset by the following tools: (a) Mian, (b) animalcules, (c) MicrobiomeAnalyst 2.0, (d) ampvis2, (e) Shiny-phyloseq and (f) Namco.


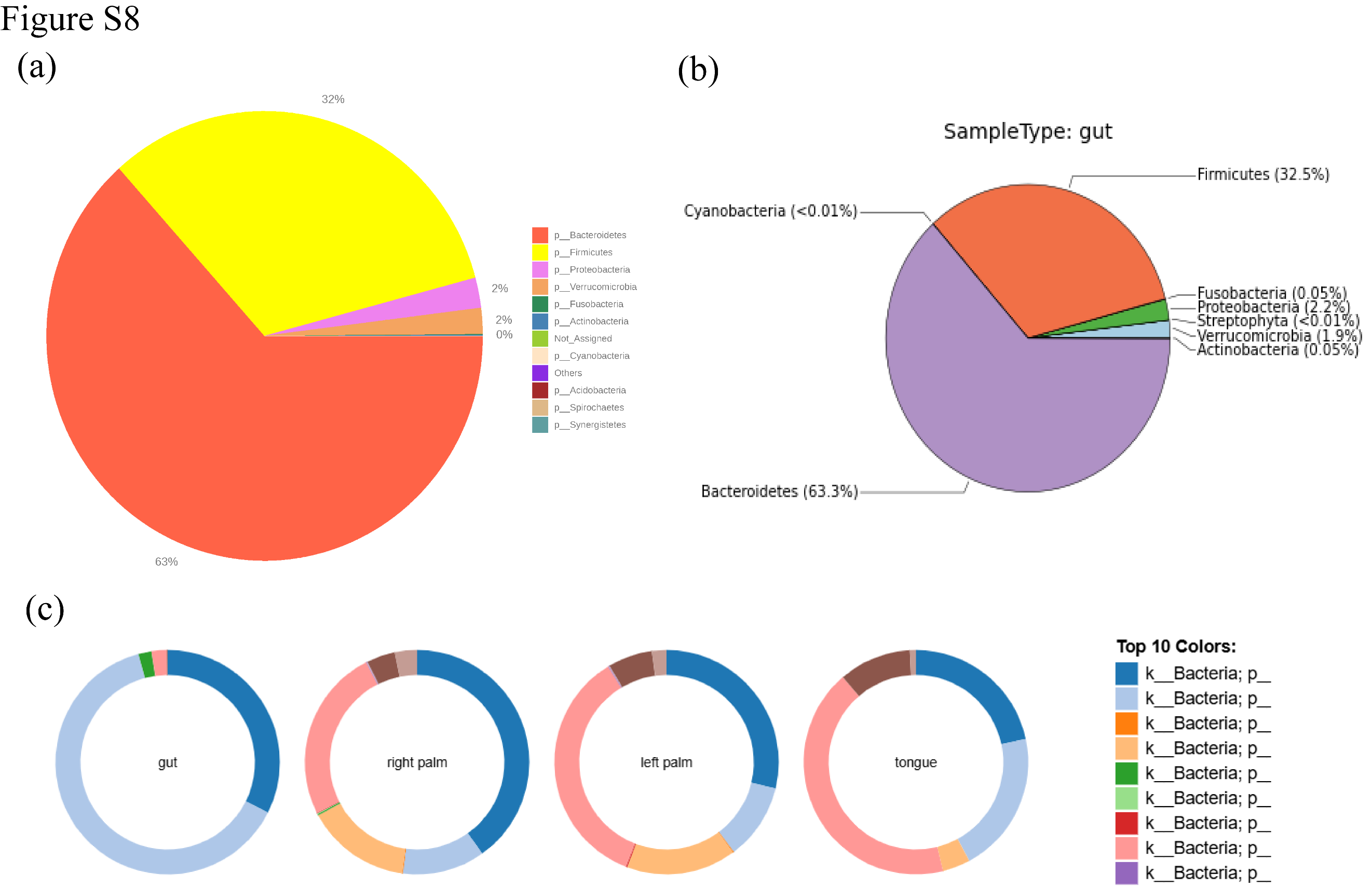


**FIGURE S11** (a) Pie chart and (b) dount of phylum-level taxonomic abundance were generated by (a) MicrobiomeAnalyst 2.0, (b) METAGENassist and (c) Mian.


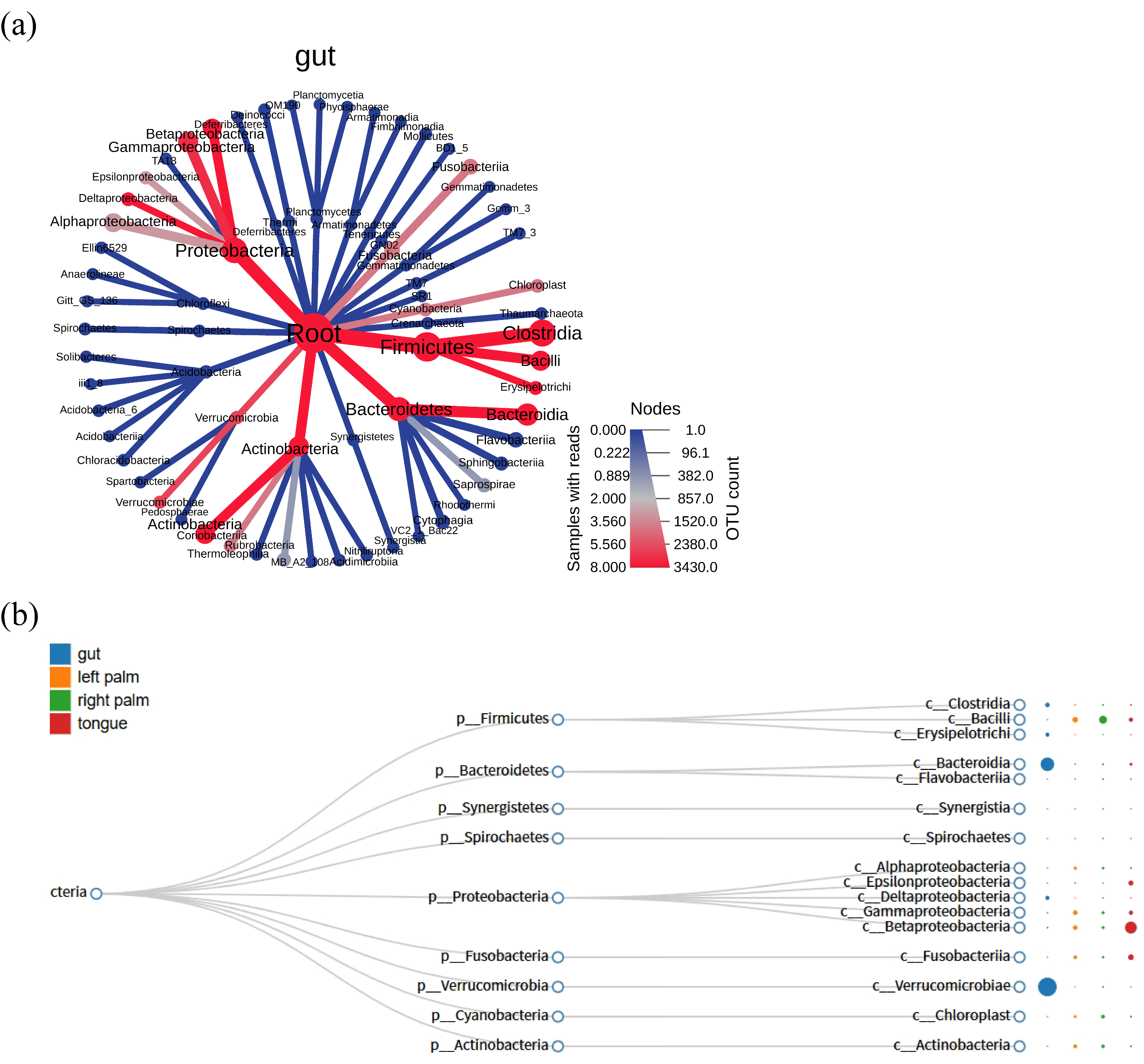


**FIGURE S12** (a) The heat tree plot generated by MicrobiomeAnalyst 2.0, color indicates the abundance and node width indicates the feature count. (b) Taxonomic tree plot produced by Mian. Node size represents either non-zero counts or the mean, median or maximum abundances of all feature-sample combinations within each taxonomic group.


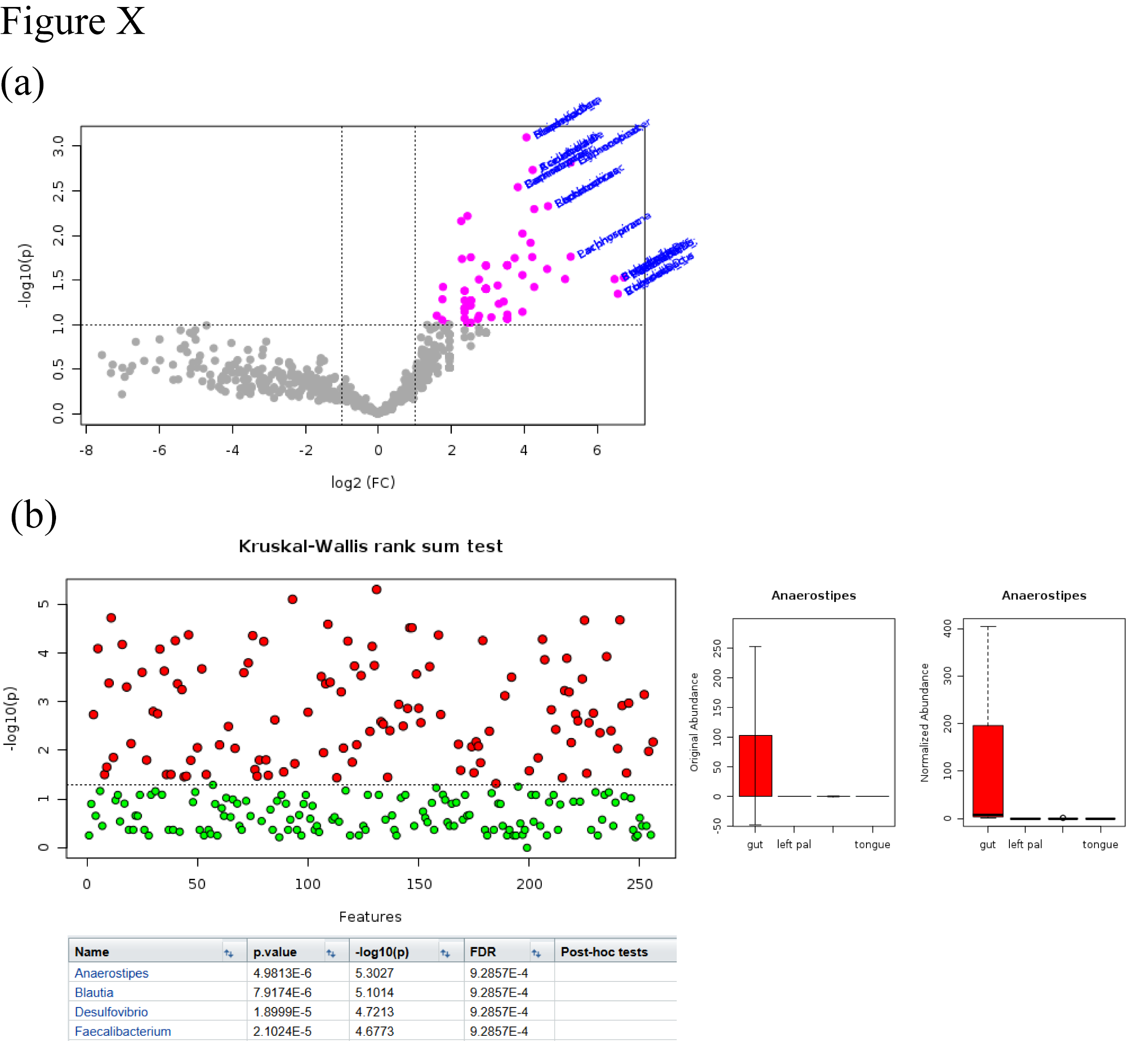


**FIGURE S13** (a) Volcano plot and (b) Manhattan plot for differential analysis generated by METAGENassist.


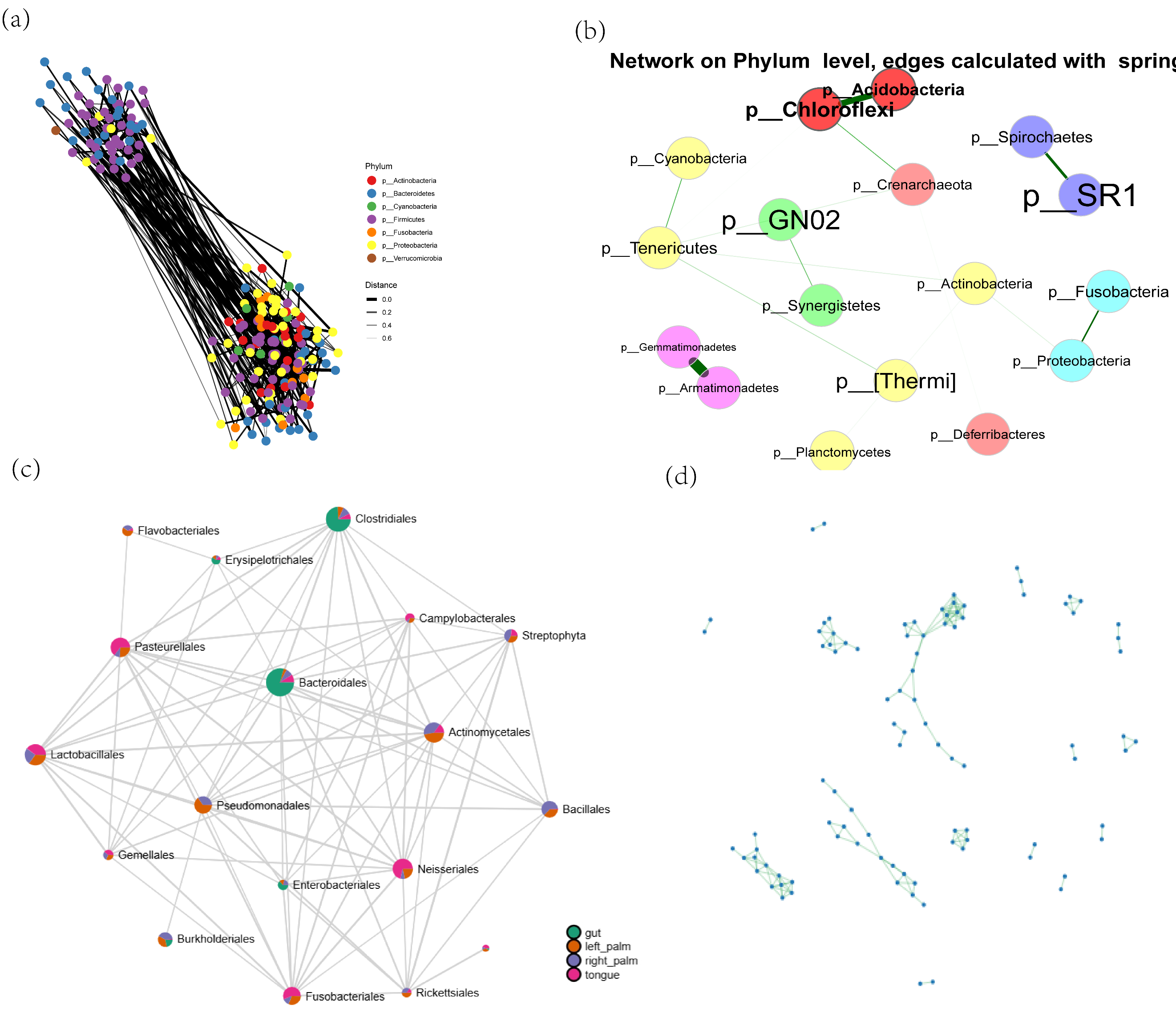


**FIGURE S14** Co-occurrence network generated by (a) Shiny-phyloseq, (b) Namco, (c) MicrobiomeAnalyst 2.0 and (d) Mian.


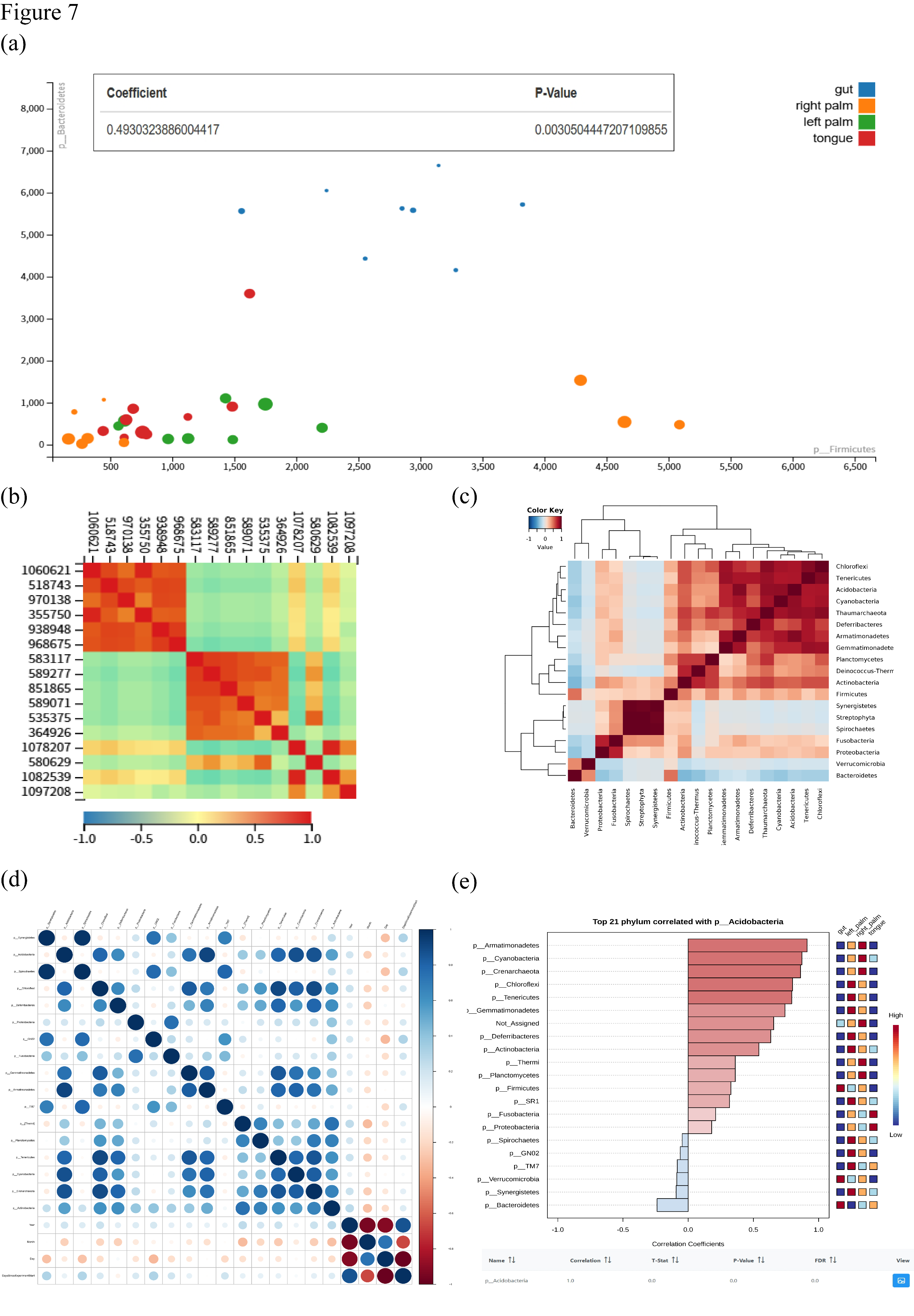


**FIGURE S15** Correlation analysis using scatter plot by (a) Mian, heatmap by (b) Mian, (c) METAGENassist, (d) Namco, barplot by (e) MicrobiomeAnalyst 2.0.


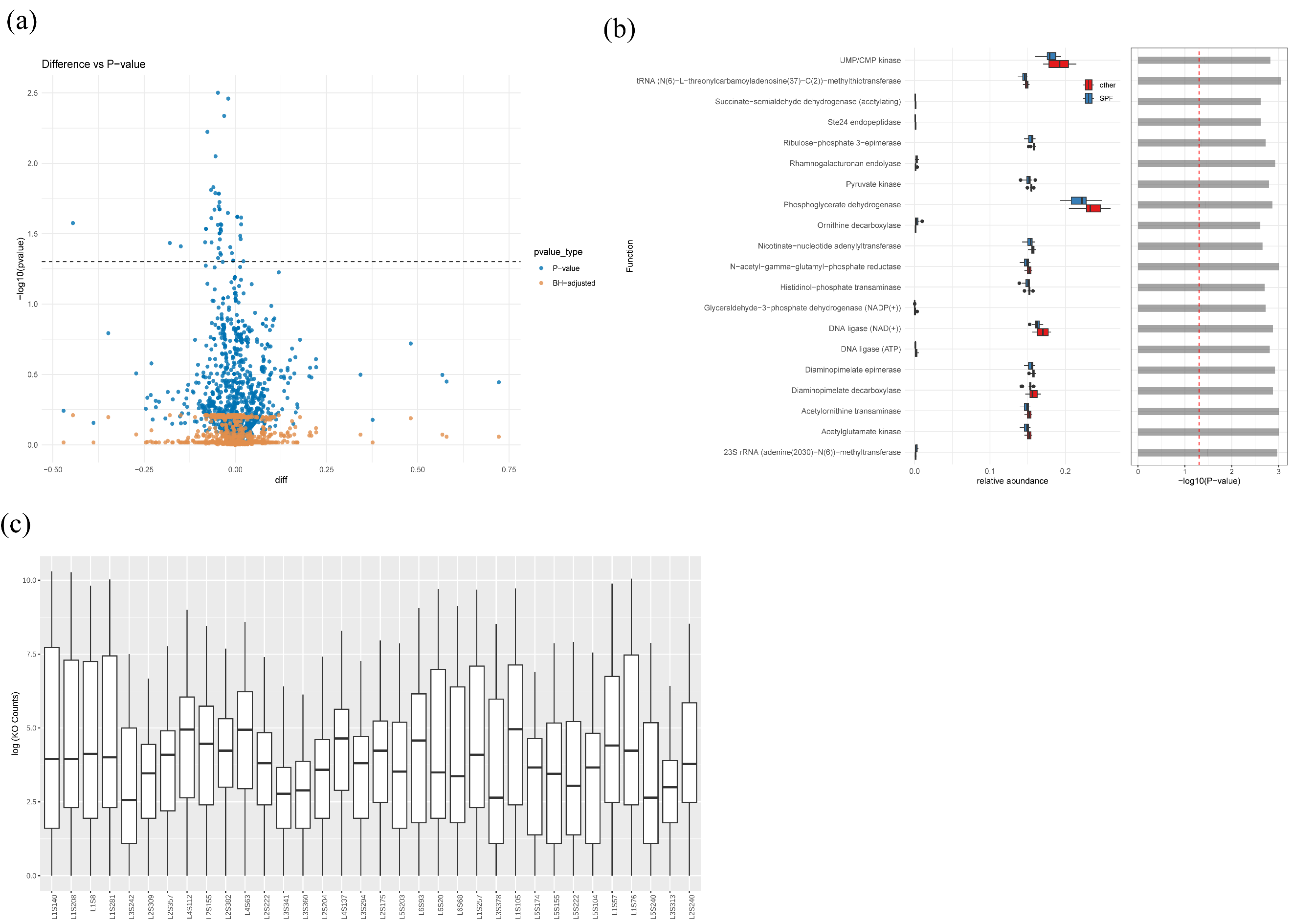


**FIGURE S16** The visualizations of functional prediction generated by PICRUSt2. (a) Volcano plot and (b) boxplot produced using Namco. (c) Boxplot generated by MicrobiomeAnalyst 2.0.


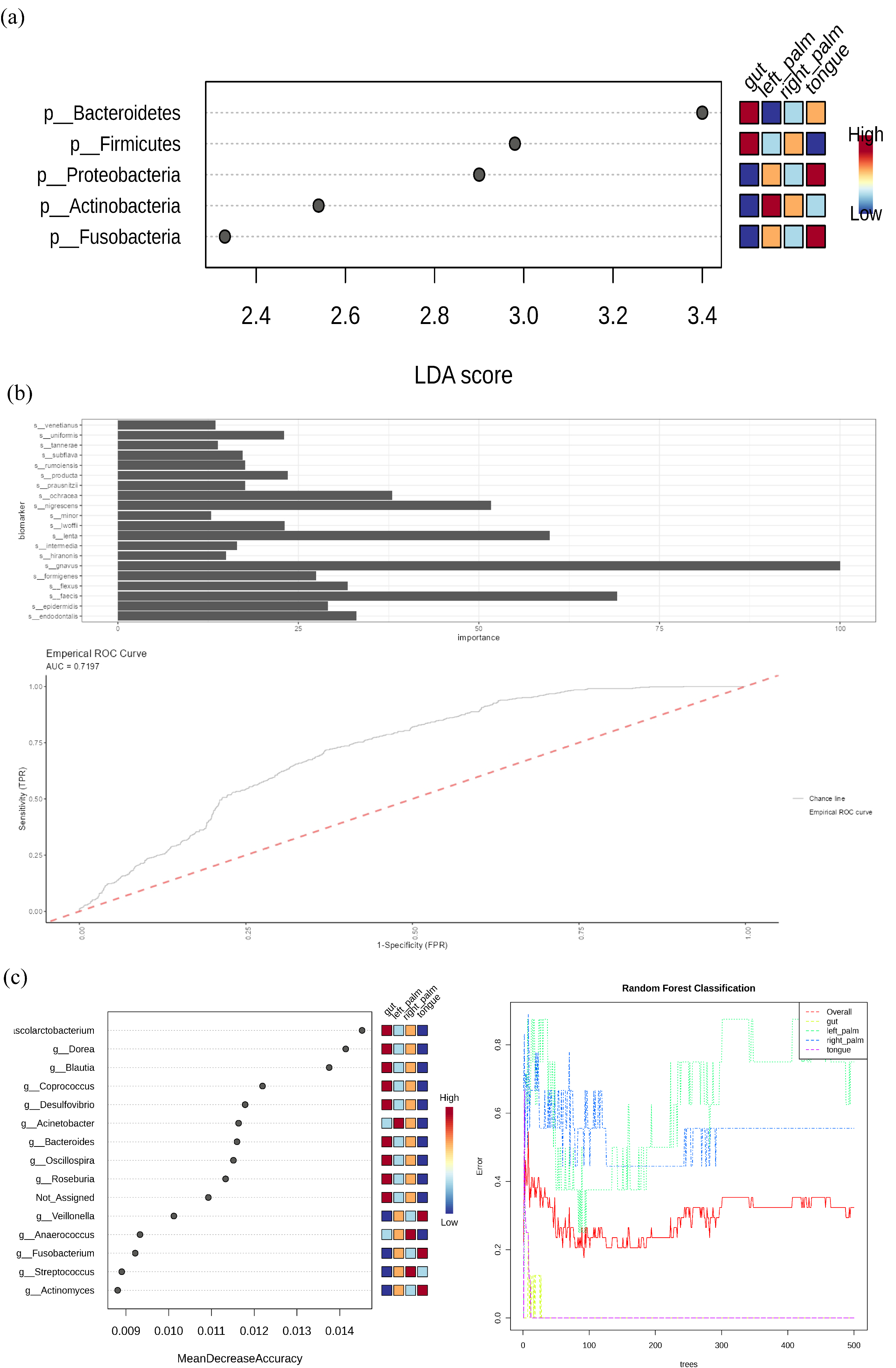


**FIGURE S17** Biomarker analysis using LEFse algorithm generated by (a) MicrobiomeAnalyst 2.0, and random forest generated by (b) animalcules and (c) MicrobiomeAnalyst 2.0.


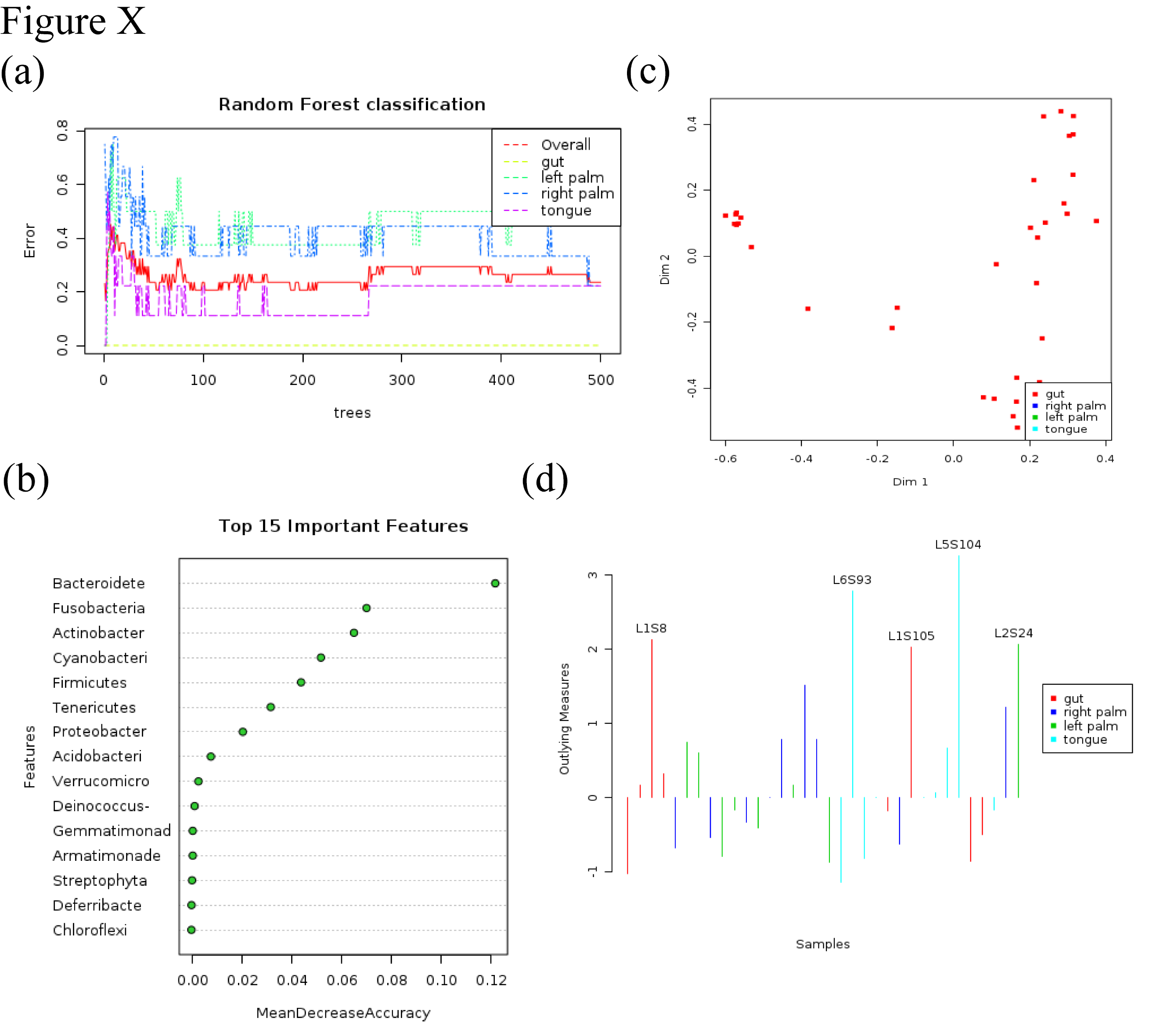


**FIGURE S18** Random forest analysis generated by METAGENassist: (a) out-of-bag (OOB) error, (b) multi-dimensional scaling (MDS), (c) feature importance and (d) outlier detection.


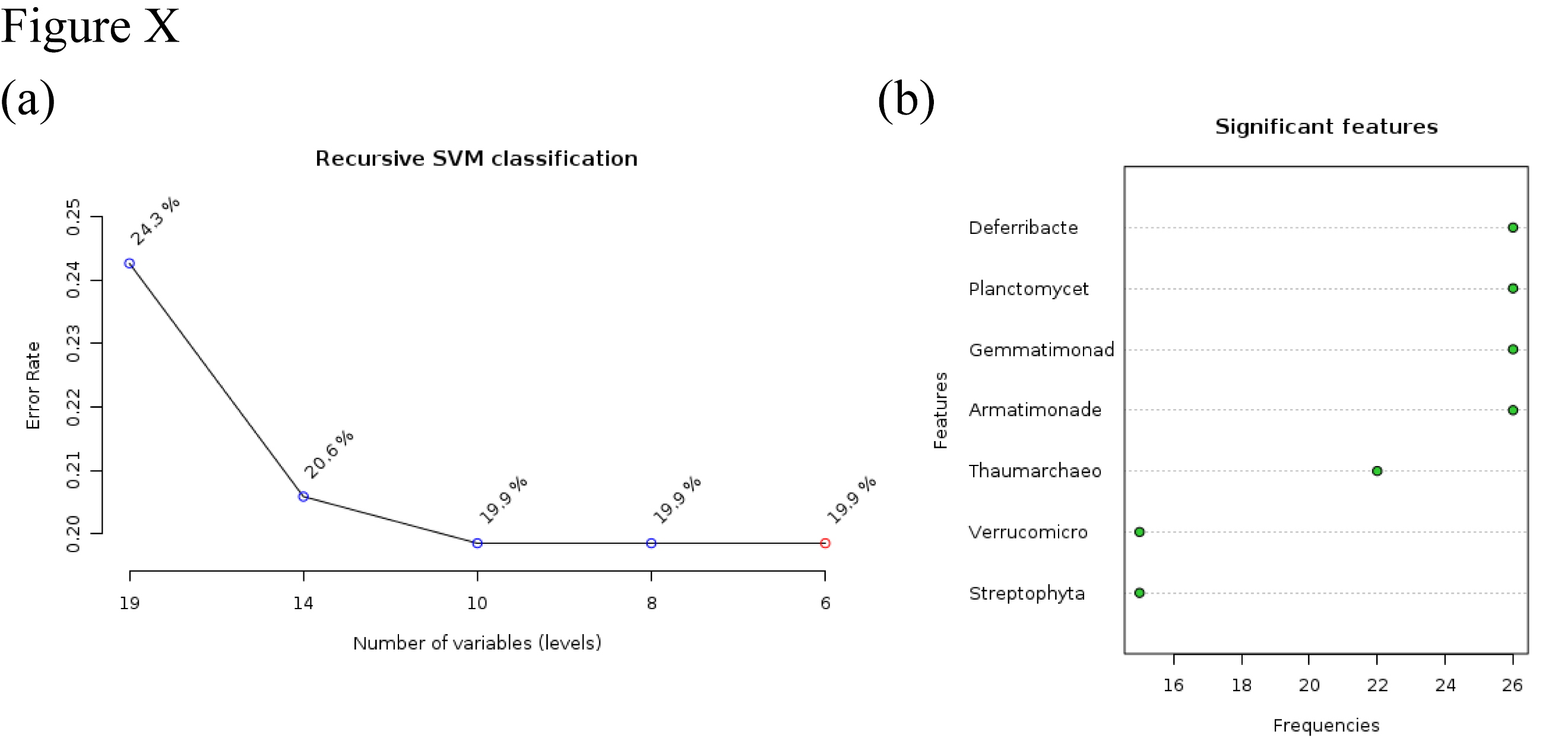


**FIGURE S19** (a) Recursive SVM classification and (b) significant features identified by SVM analysis using METAGENassist.


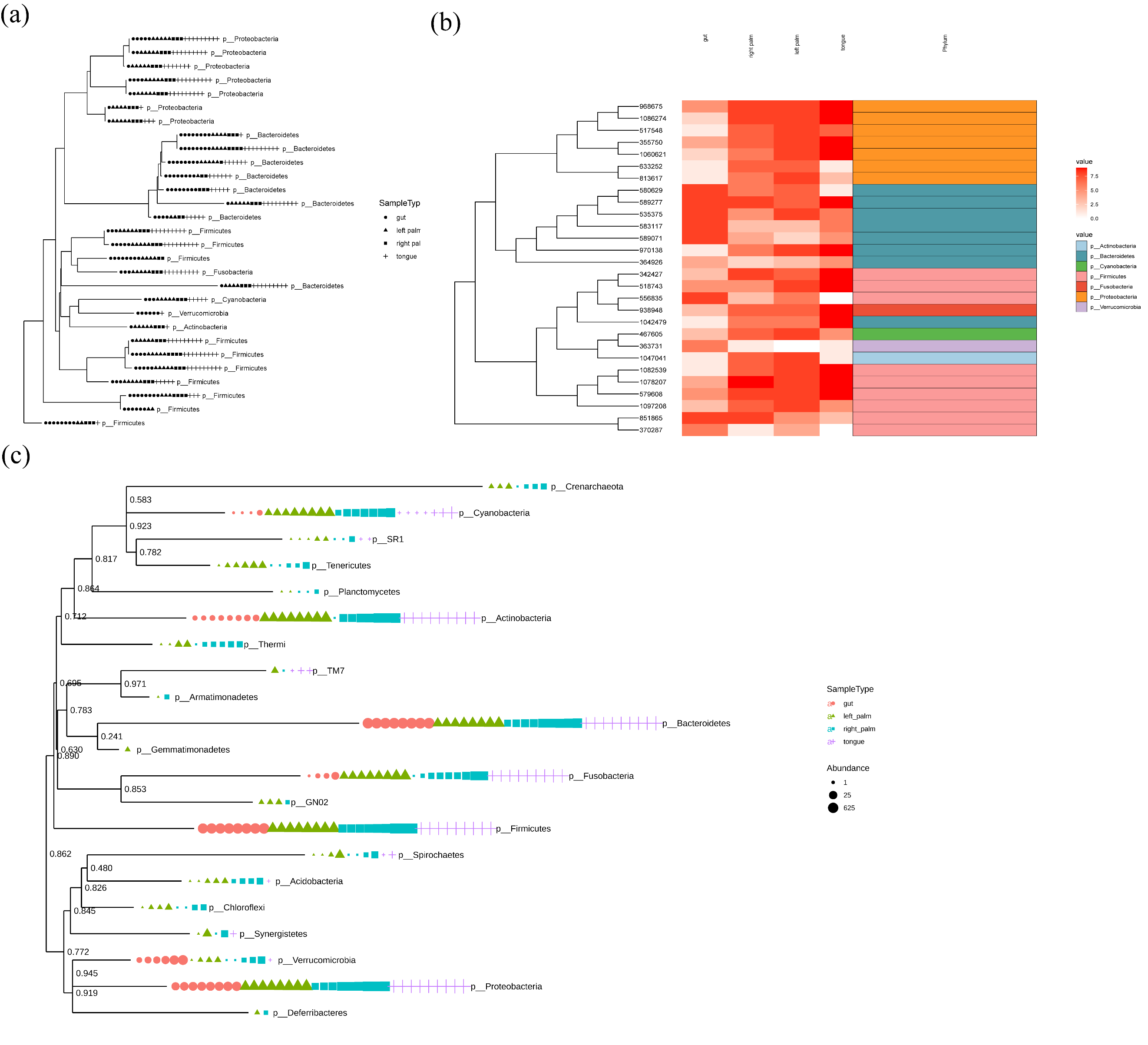


**FIGURE S20** Tree plot generated by the (a) Shiny-phyloseq, (b) Namco and (c) MicrobiomeAnalyst 2.0.
