# Supplementary material for: A comprehensive comparison of web-based tools for amplicon-metagenomic analysis
Source: Front Microbiol. 2026 Jan 6;16:1711000. doi: 10.3389/fmicb.2025.1711000 (PMC12815881; doi:10.3389/fmicb.2025.1711000)
Supplement: Supplementary file 1 [file Supplementary_file_1.zip › Input data generation using R scripts for tool testing.DOCX]

**The input data generation using R scripts for tool testing.**

Despite the availability of multiple data types available among tools, we selected the count table as the standard input format for two main reasons: the count table can be (1) easily opened and edited with common text-editing software such as Excel, facilitating users access and modification; (2) conveniently generated from the widely used phyloseq object. Specifically, input data produced by various pipelines (e.g., QIIME, mothur) can be imported into phyloseq object, from which the required individual files can be efficiently generated in R. The phyloseq object (the *caporaso* dataset included in the **microbiomeMarker** package in this study) was converted into a microeco object using the *phyloseq2meco* function from the **file2meco package**. All required tables were then exported using the *object$save_table* function in the **microeco** package. The exported files were subsequently reformatted into the input data types required by each tool through standard R processing steps. The final input data for downstream analyses consisted of individual files, including a feature table, annotation table, metadata table and an optional tree file, or alternatively a complete phyloseq object. For MicrobiomeAnalyst 2.0, the column names in the feature table, sample table and annotation table must be modified to #NAME, #NAME and #TAXONOMY, respectively. The detailed R scripts for converting a phyloseq object into the appropriate individual files required by several web-based tools were provided below.

1. Shiny-Phyloseq

***#1. data transformation***

***library(microbiomeMarker)***

***data(caporaso)***

***ps_phy<-caporaso;ps_phy***

***path<-"D:/" #setting your path***

***save(ps_phy,file = file.path(path, "ps_phy.RData"))***

***#2. importing input data***

***# install.packages("shiny")***

***shiny::runGitHub("shiny-phyloseq","joey711")***

1. animalcules

***#1. data transformation***

***library(microeco)***

***library(phyloseq)***

***library(file2meco)***

***library(microbiomeMarker)***

***data(caporaso)***

***ps<-caporaso;ps***

***meco<-phyloseq2meco(ps)***

***path<-"D:/" #setting your path***

***meco$save_table(dirpath = path, sep = "\t",quote = F)***

***#2. importing input data***

***# if (!requireNamespace("devtools", quietly=TRUE))***

***# install.packages("devtools")***

***# devtools::install_github("wejlab/animalcules")***

***library(animalcules)***

***run_animalcules()***

1. wiSDOM

***#1. data transformation***

***library(microbiomeMarker)***

***library(phyloseq)***

***data(caporaso)***

***ps<-caporaso;ps***

***library(metagMisc)***

***otu_tax_table <- phyloseq_to_df(ps, addtax = TRUE)***

***prefixes <- paste0("D_", 0:6, "__")***

***cols_to_modify <- 2:8***

***# exporting otutable and metadata***

***# exporting otutable***

***otu_tax_table[cols_to_modify] <- Map(function(col, prefix) paste0(prefix, col),***

***otu_tax_table[cols_to_modify], prefixes)***

***otu_tax_table$ID <- apply(otu_tax_table[, 2:8], 1, function(x) paste(x, collapse = ";"))***

***otu_tax_table$Taxonomy <- apply(otu_tax_table[, c(ncol(otu_tax_table),1)], 1, function(x) paste(x, collapse = " "))***

***otu_tax_table <- otu_tax_table[, c(ncol(otu_tax_table),9:(ncol(otu_tax_table)-2))]***

***write.table(otu_tax_table,"D:/otu_tax_tab.txt",quote = F,sep='\t',row.names = F,col.names = T)***

***# exporting metadata (only keeping one column)***

***metadata<-as.data.frame(as.matrix(sample_data(ps)));metadata***

***class(metadata)***

***library(tibble)***

***metadata<-rownames_to_column(metadata,var = "ID");metadata***

***metadata<- metadata[,1:2] #Based on the research objectives, keep one column (variable)***

***write.table(metadata,"D:/metadata.txt",quote = F,sep='\t',row.names = F,col.names = F)***

***#2. importing input data***

***# install.packages('shiny')***

***library(shiny)***

***shiny::runGitHub('wiSDOM','lunching')***

1. Mian

***#1. data transformation***

***library(microeco)***

***library(phyloseq)***

***library(file2meco)***

***library(microbiomeMarker)***

***data(caporaso)***

***ps<-caporaso;ps***

***# exporting otutab***

***library(tibble)***

***otu<-t(as.data.frame((otu_table(ps))))***

***otu<-as.data.frame(otu)***

***otu<-rownames_to_column(otu,var = "Sample Labels")***

***View(otu)***

***write.table(otu,"D:/otutab.txt",quote = F,sep='\t',row.names = F,col.names = T)***

***# exporting taxtab***

***tax<-as.data.frame(tax_table(ps))***

***View(tax)***

***write.table(tax,"D:/taxtab.txt",quote = F,sep='\t',row.names = T,col.names = F)***

***# adding "OTU Taxonomy" in first row of file named "taxtab.txt"***

***# exporting metadata***

***metadata<-as.data.frame(as.matrix(sample_data(ps)))***

***View(metadata)***

***class(metadata)***

***metadata<-rownames_to_column(metadata,var = "SampleID");metadata***

***write.table(metadata,"D:/metadata.txt",quote = F,sep='\t',row.names = F,col.names = T)***

1. MiCloud

***library(microbiomeMarker)***

***data(caporaso)***

***ps<-caporaso;ps***

***saveRDS(ps,"D:/ps.rds")***

1. Namco

***library(phyloseq)***

***library(file2meco)***

***library(microbiomeMarker)***

***data(caporaso)***

***ps<-caporaso;ps***

***meco<-phyloseq2meco(ps)***

***path<-"D:/"***

***meco$save_table(dirpath = path, sep = "\t",quote = F)***

1. MicrobiomeAnalyst 2.0

***library(phyloseq)***

***library(file2meco)***

***library(microbiomeMarker)***

***data(caporaso)***

***ps<-caporaso;ps***

***meco<-phyloseq2meco(ps)***

***meco$save_table(dirpath = "D:/", sep = ",",quote = F)***

***# NOTE: The ID in the feature_table has been modified to #NAME, in the sample_table to sample-id, and in the tax_table to #TAXONOMY.***

1. MiPair

***library(microbiomeMarker)***

***data(caporaso)***

***ps<-caporaso;ps***

***saveRDS(ps,"D:/ps.rds")***

1. misurv

***library(microbiomeMarker)***

***data(caporaso)***

***ps<-caporaso;ps***

***saveRDS(ps,"D:/ps.rds")***

1. ampvis2

***# loading data***

***library(microeco)***

***library(phyloseq)***

***library(file2meco)***

***library(microbiomeMarker)***

***data(caporaso)***

***ps<-caporaso;ps***

***# exporting otutable and metadata***

***# exporting otutable***

***library(metagMisc)***

***otu_tax_table <- phyloseq_to_df(ps, addtax = TRUE)***

***class(otu_tax_table)***

***otu_tax_table <- otu_tax_table[, c(1, 9:ncol(otu_tax_table), 2:8)]***

***write.table(otu_tax_table,"D:/otu_tax_table.txt",quote = F,sep=';',row.names = F,col.names = T)***

***# exporting metadata***

***metadata<-as.data.frame(as.matrix(sample_data(ps)));metadata***

***class(metadata)***

***library(tibble)***

***metadata<-rownames_to_column(metadata,var = "SampleID");metadata***

***write.table(metadata,"D:/metadata.txt",quote = F,sep='\t',row.names = F,col.names = T)***

（11）METAGENassist

***#1. data transformation***

***library(microbiomeMarker)***

***library(phyloseq)***

***data(caporaso)***

***ps<-caporaso;ps***

***library(metagMisc)***

***otu_tax_table <- phyloseq_to_df(ps, addtax = TRUE)***

***prefixes <- paste0("D_", 0:6, "__")***

***cols_to_modify <- 2:8***

***# exporting otutable and metadata***

***# exporting otutable***

***otu_tax_table[cols_to_modify] <- Map(function(col, prefix) paste0(prefix, col),***

***otu_tax_table[cols_to_modify], prefixes)***

***otu_tax_table$ID <- apply(otu_tax_table[, 2:8], 1, function(x) paste(x, collapse = ";"))***

***otu_tax_table$SampleID <- apply(otu_tax_table[, c(ncol(otu_tax_table),1)], 1, function(x) paste(x, collapse = " "))***

***otu_tax_table <- otu_tax_table[, c(ncol(otu_tax_table),9:(ncol(otu_tax_table)-2))]***

***write.table(otu_tax_table, "D:/otu_tax_tab.csv", quote = FALSE, sep = ",", row.names = FALSE, col.names = TRUE)***

***# exporting metadata (only keeping one column)***

***metadata<-as.data.frame(as.matrix(sample_data(ps)));metadata***

***class(metadata)***

***library(tibble)***

***metadata<-rownames_to_column(metadata,var = "SampleID");metadata***

***write.table(metadata,"D:/metadata.csv",quote = F,sep=',',row.names = F,col.names = T)***
